# Assessing Simultaneous Infection with Multiple Pathogens via Group Testing with Imperfect Multiplex Assays

**DOI:** 10.1007/s13253-025-00711-8

**Published:** 2025-09-18

**Authors:** Stella Self, Melissa Nolan, Kayla Bramlett, Kia Zellars, Brian H. Herrin, Christopher S. McMahan

**Affiliations:** Arnold School of Public Health, University of South Carolina, 921 Assembly Street, Columbia, SC 29208, USA; Arnold School of Public Health, University of South Carolina, 921 Assembly Street, Columbia, SC 29208, USA; Arnold School of Public Health, University of South Carolina, 921 Assembly Street, Columbia, SC 29208, USA; Arnold School of Public Health, University of South Carolina, 921 Assembly Street, Columbia, SC 29208, USA; Department of Diagnositc Medicine, College of Veterinary Medicine, 1700 Denison Avenue, Manhattan, KS 6605, USA; School of Mathematical and Statistical Sciences, Clemson University, Martin Hall, Clemson, SC 29631, USA

**Keywords:** Pooled testing, Group testing, Imperfect multiplex assays, Optimal pool size

## Abstract

Pooled testing procedures involve physically combining biomaterial from multiple individuals and testing the combined specimen for the presence of infection. Provided the prevalence of infection is relatively low, pooled testing allows one to screen many individuals at a fraction of the cost of traditional individual testing and has been widely used to screen both humans and animals for infection. Multiplex assays further increase efficiency by simultaneously screening for multiple pathogens. However, such assays are often imperfect, rendering both false-positive and false-negative results. In this work, we develop a means of estimating the prevalence of co-infections from imperfect multiplex pooled testing data for any pool size and any number of pathogens. Our approach uses an expectation–maximization (EM) algorithm to estimate the infection probabilities and uses Louis’s method to estimate the associated variance–covariance matrix. We provide a means of determining which pool size which minimizes the variance of the estimated marginal or co-infection prevalence. We also present a hypothesis test for determining if infections are mutually independent. We validate our approach with an extensive simulation study and then apply it to a pooled testing data from a multiplex assay for four tick-borne pathogens.

## 1. INTRODUCTION

Tick-borne diseases such as Lyme disease and Rocky Mountain spotted fever are a significant public health burden in the United States (U.S.). Many tick-borne diseases are curable if promptly treated with antibiotics. However, initial symptoms are often non-specific (e.g., fever, myalgia, and headache), and laboratory tests can be unreliable in the early stages of infection when treatment is most effective. Prompt diagnosis depends heavily on recognizing a patient’s potential for exposure to an infected tick, making tick surveillance critical for public health. These surveillance efforts typically involve collecting ticks and testing them for disease causing pathogens. Climate change and other factors are causing the geographic range of ticks and pathogens to shift, necessitating ongoing surveillance efforts.

Ticks are commonly co-infected with multiple pathogens, and relationships between infecting pathogens are diverse and poorly understood (Cutler et al. 2021; Sanchez-Vicente and Tokarz 2023). Co-infections with some pathogens such as *Babesia microti* and *Borrelia burgdorferi* occur more often than would be expected to occur if pathogens were infecting ticks independently Hersh et al. (2014). Co-infections with other pathogens, such as *Rickettsia amblyommatis* and *Rickettsia parkeri*, occur less often than expected under independence Gual-Gonzalez et al. (2024). Understanding co-infection dynamics is important for public health, as higher numbers of co-infected ticks increase the risk of patients concurrently contracting multiple infections. In this study, ticks were collected from state parks and animal shelters across South Carolina and tested for a variety of pathogens, including *Anaplasma* spp., *Borrelia* spp., *Ehrlichia* spp., and spotted fever group *Rickettsia* spp. Our goal is to better understand the prevalence of pathogen infection and the relationships between co-infecting pathogens in South Carolina ticks.

In the U.S., state public health agencies are typically responsible for vector surveillance efforts. Such efforts often involve testing a large number of vectors for multiple pathogens with limited resources. Group/pooled testing is a promising approach for reducing the time and testing costs required for vector surveillance. Under a group testing protocol, biomaterial from multiple vectors is physically combined into a pooled specimen, which is then tested for infection. Using group testing to estimate the prevalence of infection in vectors presents a number of statistical challenges which have received considerable attention (Thompson 1962; LE 1981; Sobel and Elashoff 1975; Chen and Swallow 1990). More recently, Hepworth and Biggerstaff have considered the problem of bias correction for the estimated prevalence for perfect Hepworth and Biggerstaff (2017) and imperfect group testing Hepworth and Biggerstaff (2020). Group testing has been successfully used to conduct surveillance for a number of pathogens, including West Nile virus (KA Bernard and LD 2001; Burkhalter et al. 2014), dengue virus (Voge et al. 2013), zika virus (Thangamani et al. 2016), *Rickettsia* spp. (Polsomboon et al. 2017), *Borrelia* spp.(Kovryha et al. 2021), and *Anaplasma* spp (Kachrimanidou et al. 2011).

In addition to group testing, the use of multiplex assays can further increase the efficiency of vector surveillance. Hughes-Oliver and Rosenberger considers the problem of prevalence estimation for perfect multiplex assays, and Tebbs et al. extends this approach to imperfect assays, though both consider only two infections in their multiplex. In this work, we develop an approach to estimate the prevalence of infections and co-infections from imperfect multiplex pool testing data for an arbitrary number of pathogens when the sensitivity and specificity of the assay are known. We also propose a hypothesis testing procedure to determine if pathogens exhibit independent co-infection patterns versus symbiotic or inhibitory patterns, that is, we can determine if infection with a particular pathogen changes the probability that the vector is also infected with other pathogen(s). To maximize the efficiency of our multiplex group testing protocol, we also develop a method for determining the pool size which minimizes the variance of any smooth function of the infection/co-infection prevalences. While a number of authors have considered the question of optimal pool testing for multiplex assays Hughes-Oliver and Rosenberger (2000); Tebbs et al. (2013); Li et al. (2017); Warasi et al. (2022), to date, this work has focused on only two diseases, while our approach can be used for a multiplex of arbitrary size.

The remainder of this paper is organized as follows. Section 2 introduces a model to estimate infection and co-infection prevalence from imperfect multiplex group testing data. We provide an expectation–maximization algorithm for fitting the model and use Louis’s method to obtain the Fisher information matrix. We then introduce a hypothesis test for mutual independence among infecting pathogens and a follow-up testing procedure for specific symbiotic/inhibitory patterns. Section 3 presents an extensive simulation study validating our proposed method. In Sect. 4, we apply our method to tick surveillance data from South Carolina and demonstrate symbiotic patterns in *Rickettsia* and *Ehrlichia* spp. infections in *Amblyomma americanum* ticks. We provide concluding remarks and directions for future work in Sect. 5.

## 2. METHODOLOGY

Suppose we are screening individual vectors (ticks, mosquitoes, etc.) for K pathogens with an imperfect multiplex assay using a group testing protocol with pools of size c. We assume that each vector is assigned to exactly one pool, and no vectors are retested. Allow $Z_{j}=\left(Z_{j1},\ldots ,Z_{jK}\right)\prime$ to be the observed testing response for the jth pool with $Z_{jk}=1$ if the jth pool tested positive for the kth pathogen and 0 otherwise. Allow ${\tilde{Z}}_{j}=\left({\tilde{Z}}_{j1},\ldots ,{\tilde{Z}}_{jK}\right)\prime$ to be the latent true status of the pool; that is, ${\tilde{Z}}_{jk}=1$ if at least one vector contributing to pool j is infected with pathogen k and 0 otherwise. We assume that the assay is imperfect with sensitivity for the kth pathogen given by $Se_{k}=P\left(Z_{jk}=1|{\tilde{Z}}_{jk}=1\right)$ and specificity $Sp_{k}=P\left(Z_{jk}=0|{\tilde{Z}}_{jk}=0\right)$. Conditional on the true pool statuses, we assume that the observed testing responses are independent across pools and across pathogens, that is

$$f(Z|\tilde{Z})=\prod_{j=1}^{J}\prod_{k=1}^{K}{\left\{Se_{k}^{{\tilde{Z}}_{jk}}{\left(1-Sp_{k}\right)}^{1-{\tilde{Z}}_{jk}}\right\}}^{Z_{jk}}{\left\{{\left(1-Se_{k}\right)}^{{\tilde{Z}}_{jk}}Sp^{1-{\tilde{Z}}_{jk}}\right\}}^{1-Z_{jk}},$$

where $Z=\left(Z_{1}^{\prime },\ldots ,Z_{J}^{\prime }\right)\prime$ and $\tilde{Z}=\left({\tilde{Z}}_{1}^{\prime },\ldots ,{\tilde{Z}}_{J}^{\prime }\right)\prime$.

We are primarily interested in estimating the prevalence of infection in the underlying populations and smooth functions thereof. Here, we allow for dependence in co-infections; that is, infection with one pathogen may make it more or less likely that a vector is infected with another pathogen. Allow $𝒴=\left\{y=\left(y_{1},\ldots ,y_{K}\right)\prime :y_{k}\in \{0,1\}\right\}$ to be the $2^{K}$ possible multinomial infection statuses and allow $\delta_{y}$ to be probability that a vector in the population has infection status y, i.e., $\delta_{y}$ is the probability a vector is infected with all pathogens for which $y_{k}=1$ and not infected with any pathogens for which $y_{k}=0$. For i=1,…,cJ, allow ${\tilde{Y}}_{i}=\left({\tilde{Y}}_{i1},\ldots ,{\tilde{Y}}_{iK}\right)\prime$ to be the unobserved true infection status of the ith vector. Thus, if ${\tilde{Y}}_{i}=y$, then vector i is infected with pathogen k if and only if $y_{k}=1;P\left({\tilde{Y}}_{i}=y\right)$ is denoted by $\delta_{y}$. Noting that we can obtain the true pool statuses $\tilde{Z}$ from the vector statuses $\tilde{Y}$, and applying the law of total probability, we have

(1) $$f(Z|\delta )=\sum_{\tilde{Y}\in {𝒴}^{cJ}}f(Z|\tilde{Y},\delta )f(\tilde{Y}|\delta )$$

where ${𝒴}^{cJ}$ is the set of all possible infection statuses for the cJ vectors, that is, ${𝒴}^{cJ}$ is the set product of 𝒴 with itself cJ times. Note that we must have $\sum_{y\in 𝒴}\delta_{y}=1$, and thus, δ contains only $2^{K}-1$ free parameters with the remaining parameter equal to 1 minus the sum of the others. Without loss of generality, we let ${\left\{\delta_{y}\right\}}_{y\in 𝒴\setminus 1}$ be the set of free parameters, where 1 denotes the K-dimensional vector of 1 s.

### 2.1. Expectation–Maximization Algorithm

Direct maximization of (1) is computationally prohibitive when J is large due to the high-dimensional nature of the summation. Instead, we treat the latent infection statuses, $\tilde{Y}$, as missing data and employ an expectation–maximization (EM) algorithm to find the maximum likelihood estimator (MLE) of δ, denoted $\overset{ˆ}{\delta }$. We assume that the latent ${\tilde{Y}}_{i}’\text{s}$ independently follows a multinomial (1,δ) distribution. Viewing the ${\tilde{Y}}_{i}’\text{s}$ as missing data then yields the following augmented data likelihood:

(2) $$f(\delta |Z,\tilde{Y})=f(Z|\tilde{Y},\delta )f(\tilde{Y}|\delta )\;=\prod_{j=1}^{J}\prod_{k=1}^{K}{\left\{Se_{k}^{{\tilde{Z}}_{jk}}{\left(1-Sp_{k}\right)}^{1-{\tilde{Z}}_{jk}}\right\}}^{Z_{jk}}{\left\{{\left(1-Se_{k}\right)}^{{\tilde{Z}}_{jk}}Sp^{1-{\tilde{Z}}_{jk}}\right\}}^{1-Z_{jk}}\times \prod_{i\in {𝒫}_{j}}\left\{\prod_{y\in 𝒴\setminus 1}\delta_{y}^{I\left({\tilde{Y}}_{i}=y\right)}\right\}{\left(1-\sum_{y\in 𝒴\setminus 1}\delta_{y}\right)}^{I\left({\tilde{Y}}_{i}=1\right)},$$

where ${𝒫}_{j}$ indexes the set of vectors who contributed to pool j, and I(⋅) is the indicator function. The EM algorithm proceeds by iterating between an expectation (E)-step and a maximization (M)-step, as described below.

For the E-step, we first define the conditional expectation of the natural log of (2) given the current parameter values $\delta^{\left(t\right)}$, and the observed pool responses Z denoted by

(3) $$Q(\delta |\delta^{\left(t\right)})=E\left[\text{log}\left\{f\left(\delta |Z,\tilde{Y}\right)\right\}\right].$$

In order to compute (3), it is necessary to compute $E[I({\tilde{Y}}_{i}=y)|Z,\delta^{\left(t\right)}]$ for all y∈𝒴. For vector i in pool j, we have the following closed form expression for the expectation:

$$E[I\left({\tilde{Y}}_{i}=y\right)|Z,\delta^{\left(t\right)}]=P({\tilde{Y}}_{i}=y|Z,\delta^{\left(t\right)})=d_{y}{\left(\sum_{z\in 𝒴}d_{z}\right)}^{-1},$$

where $d_{y}$ is proportional to the sum of $f(\delta |Z,\tilde{Y})$ over all values of $\tilde{Y}$ for which ${\tilde{Y}}_{i}=y$. A closed form expression for $d_{y}$ is provided in Web Appendix A.

In the M-step, we maximize $Q(\delta |\delta^{\left(t\right)})$ with respect to δ, that is, we compute

$$\delta^{\left(t+1\right)}=\underset{\delta }{\text{argmax}}\;Q\left(\delta |\delta^{\left(t\right)}\right).$$

We note that the first line of (2) is free of δ, and the second line is easily recognizable as a multinomial likelihood. Our maximization problem, therefore, has a closed form solution given by $\delta_{y}^{\left(t+1\right)}=c_{y}{\left(\sum_{z\in 𝒴}c_{z}\right)}^{-1}$ where

$$c_{y}=\sum_{i=1}^{cJ}E\left[I\left({\tilde{Y}}_{i}=y\right)|Z,\delta =\delta^{\left(t\right)}\right]$$

for each y∈𝒴. To implement our EM algorithm, we iterate the E and M steps until convergence, i.e., until $\sum_{y\in 𝒴}\left|\delta_{y}^{\left(t+1\right)}-\delta_{y}^{\left(t\right)}\right|<\epsilon$, for ϵ sufficiently small.

### 2.2. Inference

Recall that our primary goal is to determine if co-infection patterns differ from those expected under an independent distribution of pathogens. To accomplish this goal, we estimate the asymptotic covariance matrix of $\overset{ˆ}{\delta }$ by using Louis’s method to obtain the Fisher information matrix. Under standard regularity conditions, we have that

$$J^{1/2}(\overset{ˆ}{\delta }-\delta )\overset{d\;}{\to }N\left\{0,I^{-1}(\delta )\right\},$$

where I(δ) is the Fisher information matrix, and $\overset{d\;}{\to }$ denotes convergence in distribution as J tends to infinity. Note that since c is fixed, J→∞ implies N→∞. Specific regularity conditions are provided in Theorem 5.41 of (van der Vaart 1998) with the key condition being that the absolute value of the second partial derivatives of log $f(\delta |\tilde{Y})$ exist and are bounded by a some integrable, measurable function $\psi (\tilde{Y})$. These conditions are satisfied by the multinomial distribution provided that δ is on the interior of the parameter space. We apply Louis’s method to obtain

$$\hat{I(\delta )}=-\frac{\partial^{2}Q\left(\delta ,\overset{ˆ}{\delta }\right)}{\partial \delta ,\partial \delta^{\prime }}-\text{var}\left(\left.\frac{\partial f\left(\delta |Z,\tilde{Y}\right)}{\partial \delta }\right|\;\overset{ˆ}{\delta }\right).$$

Additional details for computing $\hat{I(\delta )}$ are provided in the Web Appendix A. Recall that since we have fixed $\delta_{1}=1-\sum_{y\in 𝒴\setminus 1}\delta_{y},\hat{I(\delta )}$ is a $\left(2^{K}-1\right)\times \left(2^{K}-1\right)$ matrix that does not contain entries for $\delta_{1}$. However, we can obtain $\text{var}\left(\delta_{1}\right)$ and $\text{cov}\left(\delta_{1},\delta_{y}\right)$ by applying the delta method.

### 2.3. Tests for Independence

A full exploration of potential dependence in co-infections involves computing confidence intervals for K2 combinations of infections (and possibly more, if co-infections with more than two pathogens are of interest). This introduces a multiple testing problem for K>2. To control the overall type I error rate, we present an approach for testing the null hypothesis of mutual independence in all co-infections versus the alternative hypothesis that there exists at least one set of two or more pathogens for which co-infections are not independent. More formally, we test the null hypothesis that $\delta_{y}=\prod_{k=1}^{K}\pi_{k}^{y_{k}}{\left(1-\pi_{k}\right)}^{1-y_{k}}$ for some $0\leq \pi_{1},\ldots ,\pi_{K}\leq 1$ versus the alternative that no such $\pi_{k}\text{s}$ exist.

When the probability of infection with each pathogen is independent of the others, we can fit the following simpler model:

$$f(Z|\pi )=\sum_{\tilde{y}\in {𝒴}^{cJ}}f\left(Z|\tilde{Y},\pi \right)f\left(\tilde{Y}|\pi \right)$$

where $\pi =\left(\pi_{1},\ldots ,\pi_{K}\right)\prime$ and $P\left({\tilde{Y}}_{i}=y|\pi \right)=\delta_{y}=\prod_{k=1}^{K}\pi_{k}^{y_{k}}{\left(1-\pi_{k}\right)}^{1-y_{k}}$. By viewing $\tilde{Y}$ as missing data, this model can be fit with an EM algorithm similar to that described in Sect. 2.1. In the E-step, we compute

$$Q(\pi |\pi^{\left(t\right)})=E\left[\text{log}\left\{f\left(\pi |Z,\tilde{Y}\right)\right\}\right].$$

For the M-step, the maximizer of $Q\left(\pi |\pi^{\left(t\right)}\right)$ is given by $\pi_{k}^{\left(t+1\right)}=b_{k1}/\left(b_{k1}+b_{k0}\right)$ where

$$b_{kl}=\sum_{i=1}^{cJ}E\left[I({\tilde{Y}}_{ik}=l|Z,\pi^{\left(t\right)})\right].$$

A closed form expression for $E\left[I\left({\tilde{Y}}_{ik}=l|Z,\pi^{\left(t\right)}\right)\right]$ is provided in Web Appendix A.

The likelihood ratio test can then be used to test the null hypothesis of independence (e.g., $H_{0}:\delta_{y}=\prod_{k=1}^{k}\pi_{k}^{y_{k}}{\left(1-\pi_{k}\right)}^{\left(1-y_{k}\right)}$ for some π) versus the alternative hypothesis of a general multinomial model (e.g., $H_{A}:\delta \in \left\{\delta :0\leq \delta_{y}\leq 1\right.$ for y∈𝒴 and $\sum_{y\in 𝒴}\delta_{y}=1\}$). If the null hypothesis is rejected, we can perform follow-up tests on specific co-infections as follows.

Allow $p=\left(p_{1},\ldots ,p_{K}\right)$, where $p_{k}=\sum_{y\in 𝒴:y_{k}=1}\delta_{y}$ is the marginal prevalence of infection with pathogen k. We can quantify the degree of dependence between infections with pathogens k and l with the function $d(k,l)=p_{k}p_{l}-\sum_{y\in 𝒴:y_{k}=1,y_{k}=1}\delta_{y}$. As d(k,l) is a smooth function of δ, we can use the delta method to obtain $\hat{\text{var}}[d(k,l)]$ and test if d(k,l)=0 via a Wald test. We can also construct (1-α)% Wald-type confidence interval for d(k,l) is the usual manner.

### 2.4. Determining the Optimal Pool Size

Deciding how many vectors to include in each pool is an important consideration when implementing a group testing protocol. The ideal pool size for any particular group testing application depends on a variety of factors, including the goal of testing (case identification or prevalence estimation), the retesting protocol (if any), the number of tests available, the number of specimens available, the prevalence of infection, and the accuracy of the diagnostic test. A variety of authors have considered the efficiency of various group testing protocols for singleplex assays. Liu et al. provides a formula for the pool size which minimizes the variance of the estimated prevalence for imperfect singleplex assays with known sensitivity and specificity, while Huang et al. considers the problem of finding the optimal group testing protocol for prevalence estimation when the sensitivity and specificity are unknown. Hughes-Oliver and Rosenberger develops a two-stage design for determining the optimal pool size for estimating the prevalence of multiple infections when a perfect assay is used. Tebbs et al. extends this approach to incorporate retesting information and imperfect assays, but consider only two infections, defining the optimal pool size as that which minimizes the expected number of tests.

Following Hughes-Oliver and Rosenberger, we define the optimal pool size as that which minimizes a chosen function of δ, though unlike Hughes-Oliver and Rosenberger, we do not require this function to be linear, only smooth. Due to the more complex nature of our problem, we are unable to provide a formula for the optimal pool size, but we can identify the optimal pool size via numerical simulations. As have Hughes-Oliver and Rosenberger and Tebbs et al., we assume that we have an estimate $\tilde{\delta }$ of δ obtained through prior studies or from the first stage in a two-stage sampling procedure. For pool sizes c∈{2,3,…,C} and J large, we generate J independent pool responses using $\tilde{\delta }$ and the assumed sensitivity and specificity of the test (see Sect. 3 for more details on the data generation). We then fit our method to these data, use the delta method to estimate the variance of our desired function, and select the pool size resulting in the smallest estimated variance.

### 2.5. A Note About Pool-Level Probabilities

We note here that in some applications, the probability of a *pool* having a certain infection status may be of interest. This information can be important for cost estimation when retesting of positive pools is required or when we wish to know how many negative pools we must observe to be (1-α)% confident that the population is disease free. These pool-level probabilities can be easily obtained as smooth functions of δ, allowing for estimation and inference if desired; see Web Appendix B for more information.

## 3. SIMULATION STUDY

We conducted a simulation study to assess the performance of our method. Specifically, we evaluated the ability of our method to estimate the multinomial probabilities δ and the marginal prevalences of each disease, p. We assess our method’s ability to estimate the standard deviation of the sampling distribution of these quantities by comparing estimates of the standard errors obtained from Louis’s method to the empirical standard deviation of our estimators. We also gauge the performance of the hypothesis test for independent infections described in Sect. 2.3, and the method for determining the optimal pool size provided in Sect. 2.4. We consider six different scenarios for the multinomial infection probabilities δ using K=3 pathogens.

For each combination of multinomial probabilities δ and pool size c∈{2,3,…,C}, we generated J=15,000 pool responses. This large sample size was chosen to reduce variability in the estimation of $\hat{I(\delta )}$, as assessing agreement between the estimated and empirical standard deviations of $\overset{ˆ}{\delta }$ is a key goal. To generate the data, we first generated cJ independent observations from a multinomial (1,δ) distribution. We then randomly assigned each individual to exactly one pool of size c and determined the true pool statuses $\tilde{Z}$ for each pool. Finally, for j=1,2,…,J and k=1,2,…,K, we independently generated $Z_{jk}$ from a binomial distribution with success probability given by $Se_{k}{\tilde{Z}}_{jk}+\left(1-Sp_{k}\right)\left(1-{\tilde{Z}}_{jk}\right)$ for $Se_{k}=Sp_{k}=0.99$.

The exact values of δ and p used in each scenario are provided in Table 1. In scenarios 1–3, the overall prevalence of infection with any pathogen(s) is relatively low, with approximately 88% of individuals being free of all infections. In scenarios 4–6, the overall prevalence of infection is moderate, with approximately 61% of individuals being infection free. In scenarios 1 and 4, infections with the three pathogens are independent. In scenarios 2 and 5, the marginal probabilities of infection with each pathogen are the same as in scenarios 1 and 4 (respectively), but some pathogens exhibit positive correlations, some exhibit negative correlations and some are independent. In scenarios 3 and 6, co-infections are more likely to occur than infection with any singular pathogen. We consider pool sizes of ranging from 2 to 15 for the low prevalence scenarios and 2 to 10 for the moderate prevalence scenarios and generate 100 datasets for each combination of scenario and pool size.

**Figure 1.**
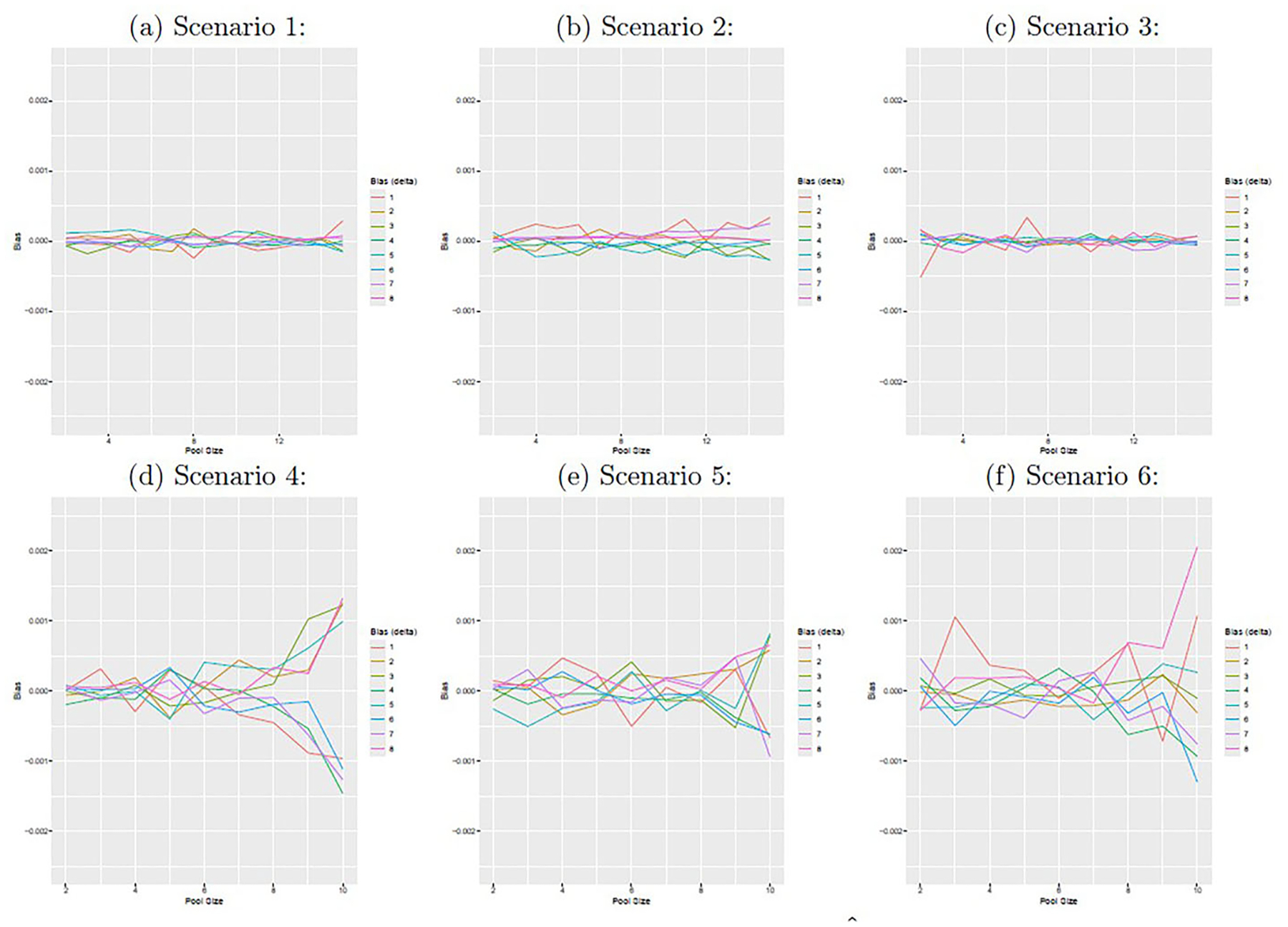
The figure displays the average empirical bias of the estimator $\overset{ˆ}{\delta }$ as a function of pool size. From 1 to 8, the position numbers correspond to y=(0,0,0),(1,0,0),(0,1,0),(1,0,1),(1,1,0),(0,1,1), and (1, 1, 1). The figure panels correspond to scenarios 1–6 from top left (1) to bottom right (6).

**Figure 2.**
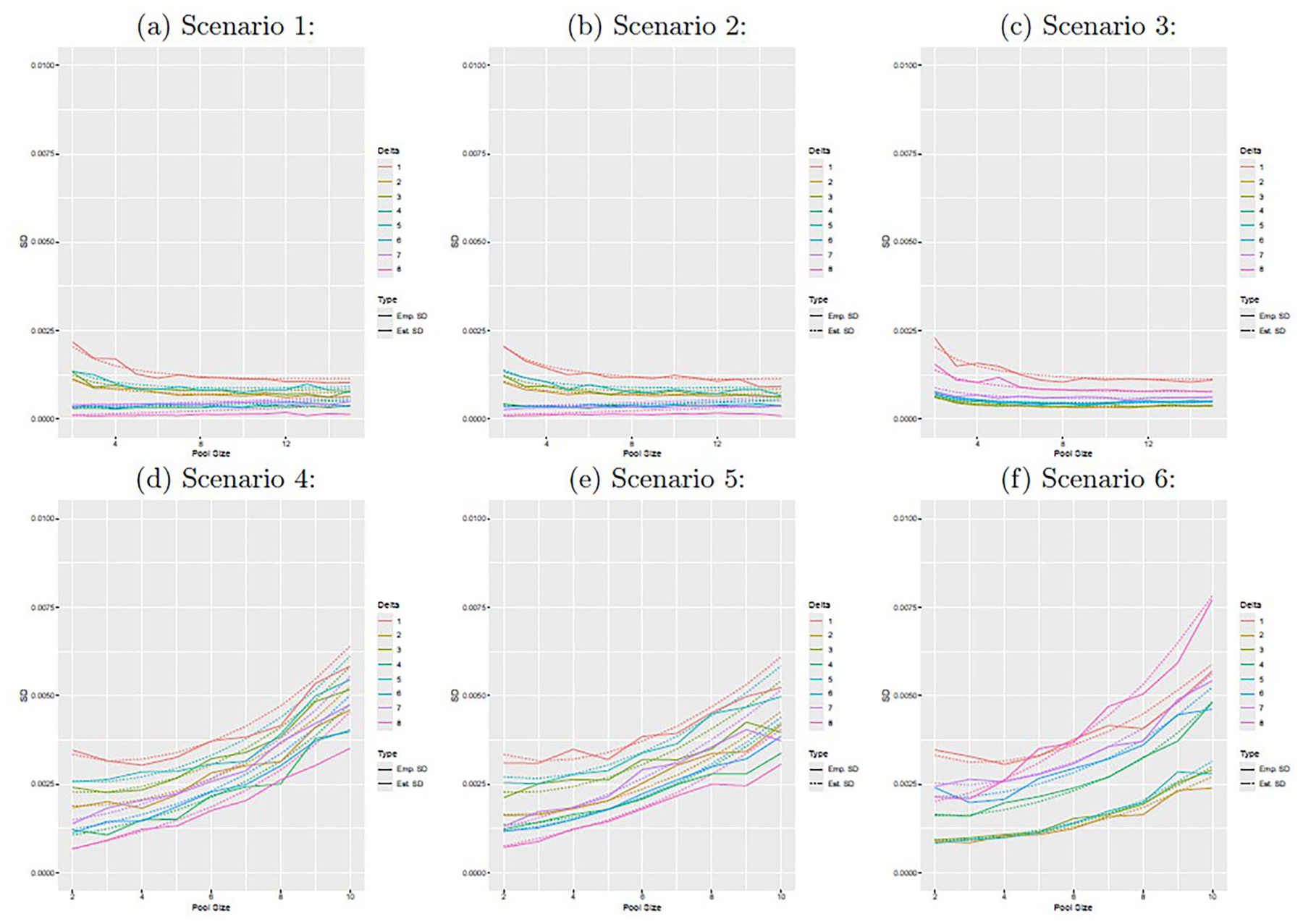
The figure displays the average estimated standard error from Louis’s method and the standard deviation of the sample of point estimators for $\overset{ˆ}{\delta }$ as a function of pool size. From 1 to 8, the position numbers correspond to y=(0,0,0),(1,0,0),(0,1,0),(1,0,1),(1,1,0),(0,1,1), and (1, 1, 1). The figure panels correspond to scenarios 1–6 from top left (1) to bottom right (6).

Figure 1 displays the average empirical bias for $\overset{ˆ}{\delta }$. Enlarged versions of each figure panel are provided in Web Figures 1–6. For the low prevalence scenarios (1–3), the bias remains roughly constant as the pool size increases from 2 to 15. For the moderate prevalence scenarios, the bias in $\overset{ˆ}{\delta }$ generally increases with the pool size. Figure 2 displays the average estimated standard errors from Louis’s method and the standard deviation of the 100 point estimators of δ. Enlarged versions of each figure panel are provided in Web Figures 7–12. These two quantities are similar for all scenarios and pool sizes, though they align more closely for smaller pool sizes, particularly in the moderate prevalence scenarios. In order to achieve this degree of agreement, we had to impose stronger convergence criteria on the EM algorithm for the moderate prevalence scenarios than for the low prevalence scenarios. We suspect that degree of agreement could be further increased with stronger convergence criteria if desired. Web Figures 13 and 14 display the empirical coverage probabilities for 95% confidence intervals for δ and the pool size which minimizes the estimated standard error of $\overset{ˆ}{\delta }$, respectively.

Web Figures 15–18 display the empirical bias, the estimated standard error and standard deviation of the point estimators, the empirical coverage probabilities of 95% confidence intervals, and the pool size minimizing the estimated standard errors (respectively) for $\overset{ˆ}{p}$. Our method estimates p accurately with reliable inference. Our estimated standard errors and standard deviations of the samples of point estimators agree with each other and with the formula provided by Liu et al. (2011) for singleplex assays.

**Figure 3.**
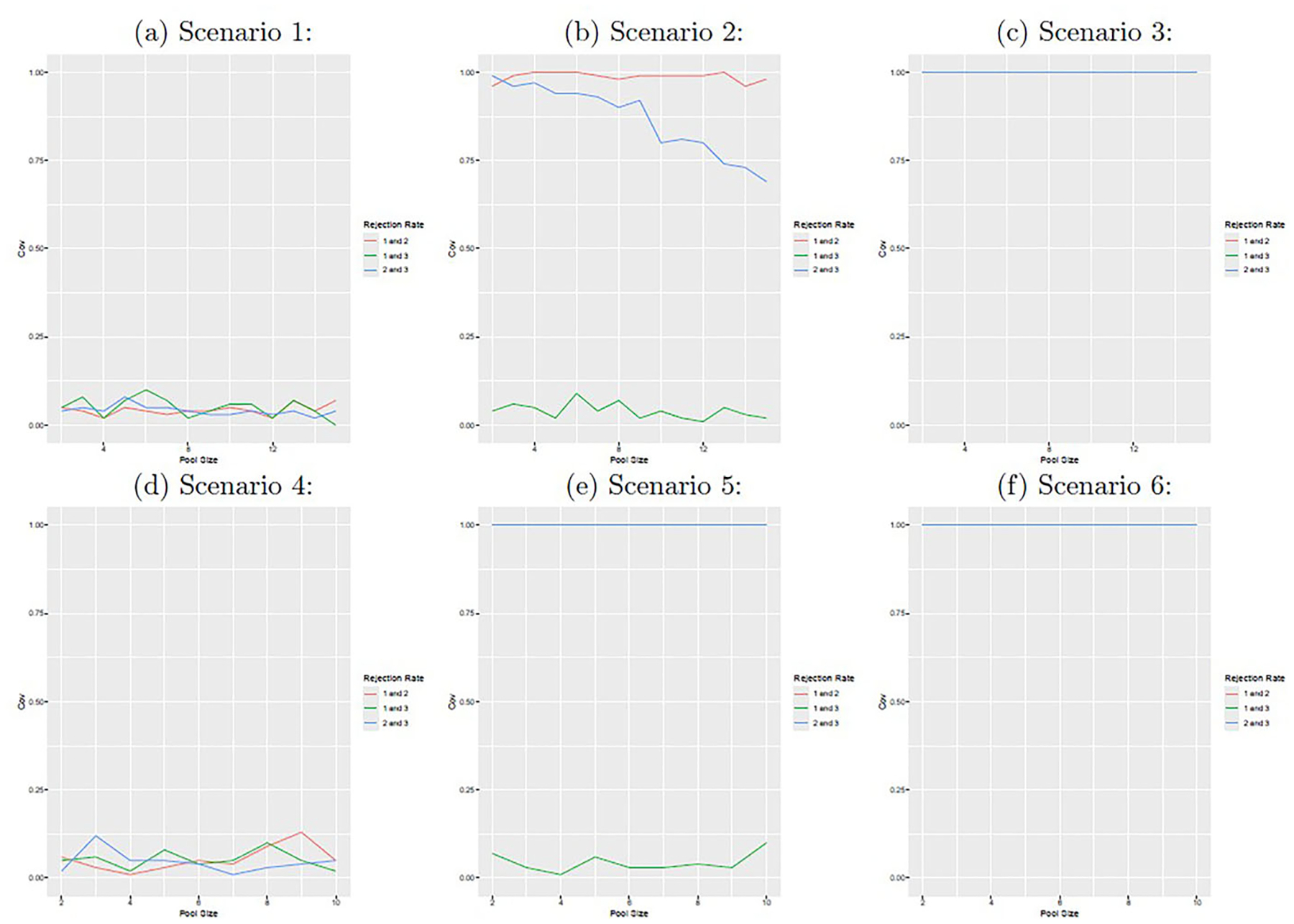
The figure displays the average empirical rejection rate for the tests for co-infection independence. The figure panels correspond to scenarios 1–6 from top left (1) to bottom right (6).

**Figure 4.**
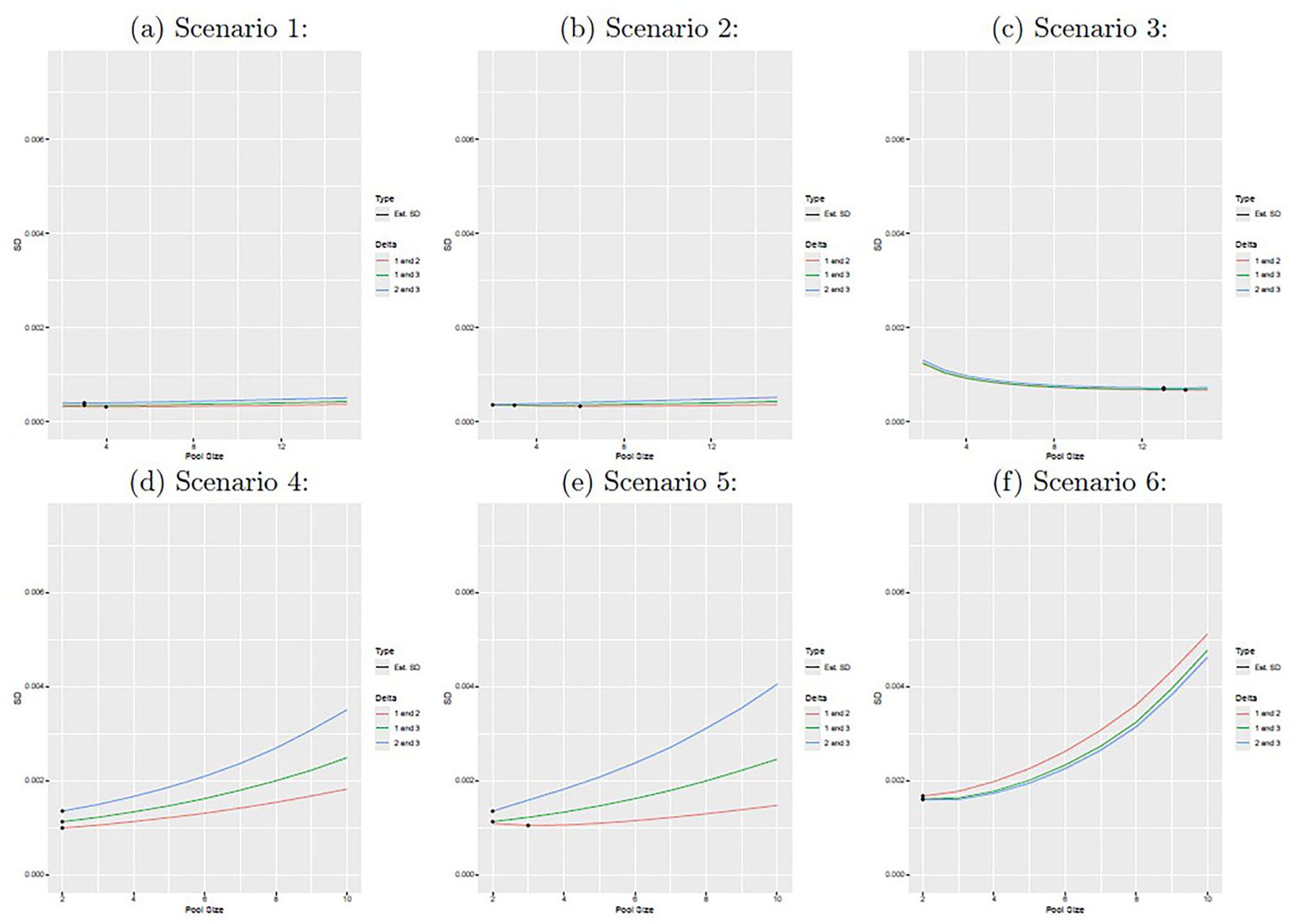
The figure displays the average estimated standard error from Louis’s method for $\overset{ˆ}{d}(1,2),\overset{ˆ}{d}(1,3)$, and $\overset{ˆ}{d}(2,3)$ as a function of pool size. The black dot indicates the pool size with the smallest standard deviation for each estimator. The figure panels correspond to scenarios 1–6 from top left (1) to bottom right (6).

Figure 3 shows the empirical rejection rate for testing if d(1,2),d(1,3), and d(2,3) are equal to 0 with a significance level of 5%. We find that that for pairs of infections which are independent (e.g., all pairs of infections in scenarios 1 and 4, and infections 1 and 3 in scenarios 2 and 4), the test maintains its nominal type I error rate with an empirical rejection rate of approximately 5% regardless of pool size. When infections are dependent, the empirical rejection rate is generally high, though the power does decline somewhat with pool size in some instances. Figure 4 displays the estimated standard deviations of $\overset{ˆ}{d}(1,2)$, $\overset{ˆ}{d}(1,3)$, and $\overset{ˆ}{d}(2,3)$ obtained from the delta method; the pool size corresponding to the lowest standard deviation is indicated with a black dot. We note that the optimal pool sizes varies depending on the infection pair under consideration.

We also conducted a simulation study to assess the performance of the test for mutual independence described in Sect. 2.3 and found that the test maintained its type I error rate under the null hypothesis and had high power under the alternative; additional details can be found in Web Appendix C.

## 4. DATA APPLICATION

Our motivating data application consists of ticks collected at various locations across South Carolina from 2021 to 2024 and tested for *Anaplasma* spp., *Borrelia* spp., *Ehrlichia* spp., and *Rickettisa* spp. infections. The original sample was diverse and included many species and life stages of ticks tested for varying combinations of pathogens in different pool sizes. For the purpose of demonstrating this methodology, we restrict our attention to adult *Amblyomma americanum* ticks tested in pools of size 3 for *Rickettsia amblyommatis*, *Rickettisa parkeri*, Panola Mountain *Ehrlichia* (PME), and *Ehrlichia ewingii*. Ticks were tested with a multiplex qPCR assay developed in-house at the Nolan Laboratory of Zoonotic and Infectious Disease at the University of South Carolina. The assay was validated using spiked control ticks from the Southeastern Center for Vector-Borne Disease and correctly identified all infected and non-ticks. For model fitting purposes, we assumed that the assay was highly accurate $\left(Se_{k}=Sp_{k}=0.99\right)$. Table 2 provides a summary of the pool-level test results.

We fit our proposed model assuming that $Se_{k}=Sp_{k}=0.99$ and applied the test for independence described in Sect. 2.3 to 191 pools consisting of three *A. americanum* ticks each. We found a likelihood ratio test statistic of $22.32>\chi_{0.95,9}=16.92$, so we rejected the null hypothesis of independence in favor of alternative hypothesis of the general multinomial model. The estimate of $\overset{ˆ}{\delta }$ from the full multinomial model is provided in Table 3.

The empirical Fisher information matrix from the full model was not invertible. Upon closer examination, we attributed this to the extremely low estimate of several of the ${\overset{ˆ}{\delta }}_{y}$ parameters. We refit the model setting $\delta_{y}=0$ for all y for which ${\overset{ˆ}{\delta }}_{y}<10^{-50}$. Note that as setting this combination of $\delta_{y}\text{s}$ to 0 violates the assumptions of the independent model, we did not retest for independent using the reduced model. However, we used a likelihood ratio test to compare this reduced model to the full model and failed to reject the null hypothesis. (Note that as the null hypothesis exists on the boundary of the parameter space, a Monte Carlo procedure was used to estimate the null distribution of the test statistic; details are provided in Web Appendix D.) We report $\overset{ˆ}{\delta }$ from the reduced model in Table 3. The Fisher information matrix from this model was invertible.

We performed a chi-squared goodness of fit test on the full, independent and reduced models. First, we compute $P\left({\tilde{Z}}_{j}=z\right)≔{\overset{ˆ}{\theta }}_{z}$ for each z∈𝒴 by applying the method described in Web Appendix B to $\overset{ˆ}{\delta }$. We then compute $P\left(Z_{j}=z\right)=\sum_{\tilde{z}}P(Z_{j}=z|{\tilde{Z}}_{j}=\tilde{z}){\overset{ˆ}{\theta }}_{\tilde{z}}≔{\overset{ˆ}{\phi }}_{z}$ for each z∈𝒴 and test the null hypothesis that the $Z_{j}\text{s}$ are an independent sample from a multinomial distribution with probability vector $\overset{ˆ}{\phi }$ versus the alternative that the multinomial probability vector is not equal to $\overset{ˆ}{\phi }$. We fail to reject the null hypothesis for the full and reduced models, with both tests having a p-value of 0.0868, indicating that these models fit the data well. For the independent model, we reject the null hypothesis with a p-value < 0.0001, indicating that the independent model is not a good fit for the data.

Since we rejected the null hypothesis of independence, we wish to determine which specific pathogens exhibit dependence in co-infections. Only two pairs of pathogens exhibited a nonzero estimated prevalence of co-infection in our reduced model: *R. parkeri*–PME and *R. amblyommatis*–*E. ewingii*. We applied the delta method to obtain $\overset{ˆ}{d}(2,3)=-0.0013$, 95% confidence interval (−0.0013, −0.0012). As this confidence interval does not contain 0, we conclude that co-infection with *R. parkeri* and PME are not independent events. As $\hat{p_{2}p_{3}}<\sum_{y\in 𝒴:y_{2}=1,y_{3}=1}{\overset{ˆ}{\delta }}_{y}$, we can further conclude that co-infection with *R. parkeri* and PME is more likely than expected under independence, and the two infections exhibit a positive association. We also used the delta method to obtain $\overset{ˆ}{d}\left(1,4\right)=-0.0046$, 95% confidence interval (−0.0047, −0.0046) and conclude that infection with *R. amblyommatis* and *E. ewingii* is also positively associated. However, we point out that for both pairs of pathogens, the prevalence of co-infection is less than 1% and the magnitude of the deviation in co-infection prevalence from that expected under independence is not large. We also performed a sensitivity analysis using $Se_{k}=Sp_{k}=0.97$; the results agreed with those of the primary analysis and are presented in Web Appendix E.

**Figure 5.**
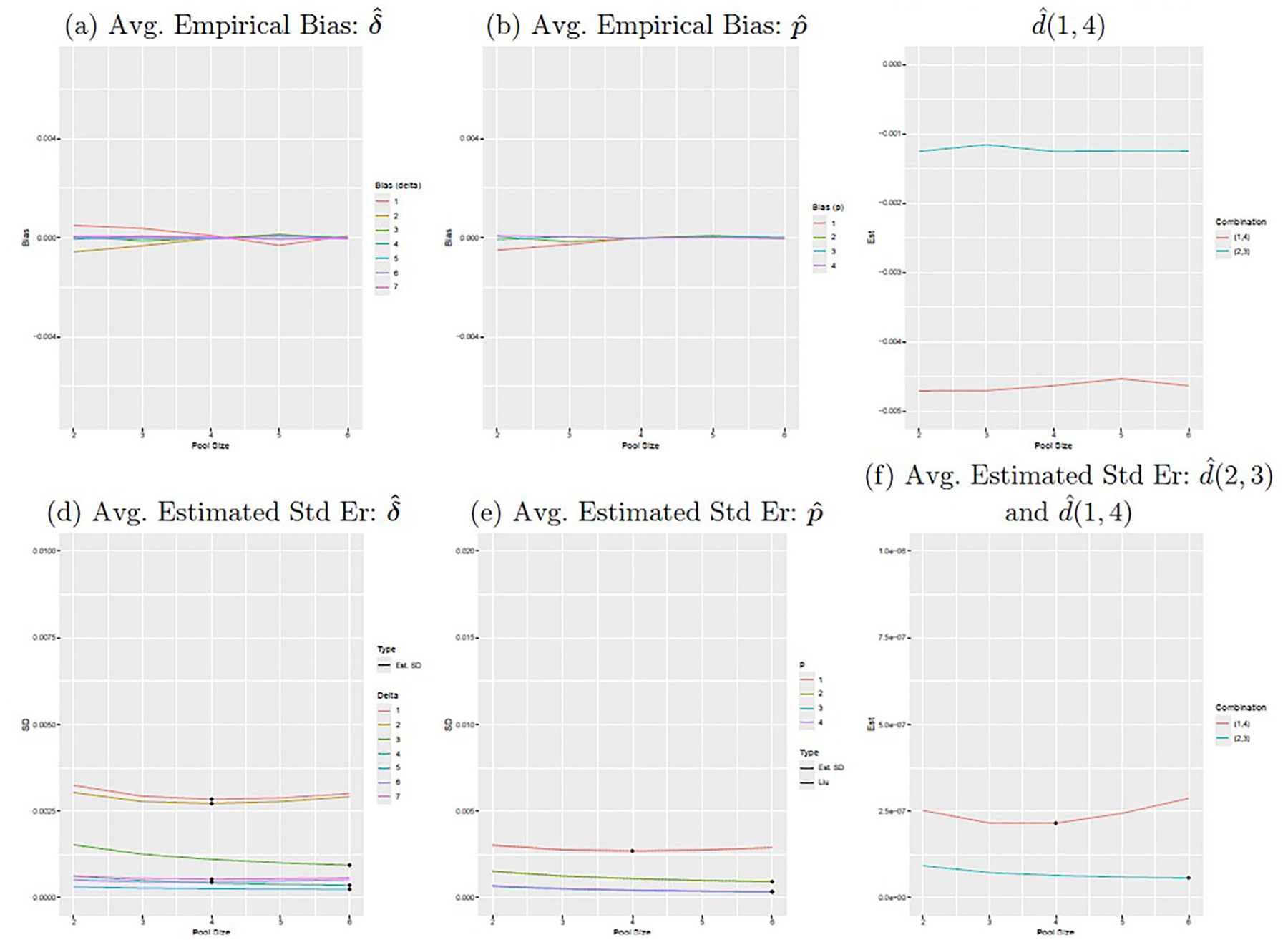
The figure displays the average empirical bias of $\overset{ˆ}{\delta }$ (top left) and $\overset{ˆ}{p}$ (top center), average estimated standard error from Louis’s method for $\overset{ˆ}{\delta }$ (bottom left) and $\overset{ˆ}{p}$ (bottom center), estimate of $\overset{ˆ}{d}(2,3)$ and $\overset{ˆ}{d}(1,4)$ (top right), and estimated standard error of $\overset{ˆ}{d}(2,3)$ and $\overset{ˆ}{d}(1,4)$ (bottom right). The position numbers for $\overset{ˆ}{\delta }$ correspond to their positions in Table 3 after removing the $\delta_{y}=0$ in the reduced model.

These data were collected as part of preliminary efforts for the ‘Group Testing is Cost Saving’ (G-TICS) study, which is an R01 funded project to develop and implement group testing methods for tick-borne disease surveillance. We wished to use these preliminary data to inform the choice of pool size for future data collected as part of the G-TICS study. To determine the optimal pool size for *A. americanum* adult ticks collected in South Carolina, we generated 100 datasets with J=15000 pool responses using the estimated value of $\overset{ˆ}{\delta }$ (reduced) in Table 3 and pool sizes ranging from 2 to 6. From these results, shown in Figure 5, we see that the optimal pool size for estimating d(1,4) is 4, while the optimal pool size for estimating d(2,3) is at least 6.

## 5. CONCLUSION

We have developed a method for estimating individual infection and co-infection prevalences from imperfect multiplex group testing data with an arbitrary pool size. This method also allows for reliable inference on the infection probabilities. We also present a test for mutual independence among pathogen infections and a follow-up testing procedure to determine which group(s) of pathogens exhibit dependent co-infection pathogens. Our method can also be used to determine the optimal pool size for imperfect multiplex group testing applications. After validating our method with an extensive simulation study, we apply it to a dataset of multiplex testing results in *A. americanum* ticks collected in South Carolina. Our method allowed us to conclusively show that co-infection with *R. parkeri*, and PME is more common that would be expected under an independent distribution of pathogens, as is co-infection with *R. amblyommatis* and *E. ewingii*.

Our method provides a number of opportunities for future work. This methodology applies to master pool testing only and would require significant modifications to incorporate group testing protocols with retesting information (e.g., Dorfman testing or array testing). While this work assumes that the sensitivity and specificity of the assay employed is known, future work could explore estimating these parameters, which would likely require the incorporation of retesting information or the translation of the method into the Bayesian framework in which informative priors could be used for the assay accuracy parameters. The computational burden of the our method increases exponentially in both the pool size and the number of pathogens, but future work may be able to reduce the computational expense. One major limitation of our approach is that it assumes the latent infection statuses are independent and identically distributed multinomial random variables. In many applications, one may wish to allow the infection probabilities to depend on covariates or to account for potential clustering among individual status (e.g., spatial clustering among ticks collected at the same locations). Clustering could be modeled by using the Dirichlet-multinomial distribution presented by Brier (1980) for modeling clustered multinomial data. As multiplex assays continue to grow in popularity, the need for group testing methodologies appropriate for such assays will continue to increase.

## Supplementary Material

Supplementary Material

The online supplementary material contains five Web Appendices. Web Appendix A contains additional details for implementing the EM algorithm and Louis’s method from Section 2. Web Appendix B describes how to obtain the probability of a pool having a certain infection status from δ. Web Appendix C contains additional simulation results. Web Appendix D provided additional details about the likelihood ratio test described in Section 4. Web Appendix E contains results from the sensitivity analysis.

Code implementing these methods is available at https://github.com/scwatson812/GroupTestingForMultiplexAssays/

Supplementary materials for this article are available at https://doi.org/10.1007/s13253-025-00711-8.

## Figures and Tables

**Table 1.** The table displays the true multinomial infection probabilities used for the simulation study (δ) and the associated marginal infection probabilities (p)

	$\delta_{\left(0,0,0\right)}$	$\delta_{\left(1,0,0\right)}$	$\delta_{\left(0,1,0\right)}$	$\delta_{\left(1,1,0\right)}$	$\delta_{\left(0,0,1\right)}$	$\delta_{\left(1,0,1\right)}$	$\delta_{\left(0,1,1\right)}$	$\delta_{\left(1,1,1\right)}$
1	0.88464	0.02736	0.03686	0.00114	0.04656	0.00144	0.00194	0.00006
2	0.88464	0.02592	0.03686	0.00258	0.04800	0.00144	0.00050	0.00006
3	0.88464	0.00500	0.00500	0.01000	0.01000	0.01000	0.02000	0.05536
4	0.61200	0.06800	0.10800	0.01200	0.15300	0.01700	0.02700	0.00300
5	0.61200	0.04800	0.10800	0.03200	0.17300	0.01700	0.00700	0.00300
6	0.61200	0.01000	0.01000	0.05000	0.01000	0.10000	0.15000	0.05800
	$p_{1}$	$p_{2}$	$p_{3}$					
1	0.03000	0.04000	0.05000					
2	0.03000	0.04000	0.05000					
3	0.08036	0.09036	0.09536					
4	0.10000	0.15000	0.20000					
5	0.10000	0.15000	0.20000					
6	0.21800	0.26800	0.31800					

**Table 2.** The table provides the percent of pools which tested positive for each combination of pathogens (right) and the marginal positivity percent for each pathogen (bottom)

	R. amblyommatis	R. parkeri	PME	E. ewingii	%
	0	0	0	0	16.75
	1	0	0	0	58.64
	0	1	0	0	13.61
	1	1	0	0	4.18
	0	0	1	0	1.57
	1	0	1	0	0.52
	0	1	1	0	1.04
	1	1	1	0	0.00
	0	0	0	1	0.52
	1	0	0	1	3.14
	0	1	0	1	0.00
	1	1	0	1	0.00
	0	0	1	1	0.00
	1	0	1	1	0.00
	0	1	1	1	0.00
	1	1	1	1	0.00
%	66.49	18.85	3.14	3.66	

**Table 3.** The table provides the estimated multinomial probabilities $\left(\overset{ˆ}{\delta }\right)$ and estimated marginal prevalences $\left(\overset{ˆ}{p}\right)$ for the South Carolina tick data

	*R. amblyommatis*	*R. parkeri*	*PME*	*E. ewingii*	$\overset{ˆ}{\delta }$ (Full)	$\overset{ˆ}{\delta }$ (Reduced)
	0	0	0	0	0.6094	0.6094
	1	0	0	0	0.3080	0.3080
	0	1	0	0	0.0651	0.0651
	1	1	0	0	< 10^−50^	0
	0	0	1	0	0.0063	0.0063
	1	0	1	0	< 10^−50^	0
	0	1	1	0	0.0018	0.0018
	1	1	1	0	< 10^−50^	0
	0	0	0	1	0.0018	0.0018
	1	0	0	1	0.0076	0.0076
	0	1	0	1	< 10^−50^	0
	1	1	0	1	< 10^−50^	0
	0	0	1	1	< 10^−50^	0
	1	0	1	1	< 10^−50^	0
	0	1	1	1	< 10^−50^	0
	1	1	1	1	< 10^−50^	0
$\overset{ˆ}{p}$ (Full)	0.3156	0.0669	0.0081	0.0094		
$\overset{ˆ}{p}$ (Reduced)	0.3156	0.0669	0.0081	0.0094		
