# Supplementary material for: Assessing Simultaneous Infection with Multiple Pathogens via Group Testing with Imperfect Multiplex Assays
Source: J Agric Biol Environ Stat. Author manuscript; Available in PMC 2026 May 2. (PMC13134653; doi:10.1007/s13253-025-00711-8)
Supplement: Supplementary Material [file NIHMS2164513-supplement-Supplementary_Material.pdf]

Supplementary Material for 'Assessing simultaneous infection with multiple pathogens via group testing with imperfect multiplex assays'

Web Appendix A

This section contains additional details for the implementation of the EM algorithm and Louis's method as described in Section 2.

Additional Details for the EM Algorithm

Recall that the E-step in the EM algorithm requires computing

$$E[I(\tilde{\boldsymbol{Y}}_i = \boldsymbol{y})|\boldsymbol{Z}, \boldsymbol{\delta}^{(t)}] = P(\tilde{\boldsymbol{Y}}_i = \boldsymbol{y}|\boldsymbol{Z}, \boldsymbol{\delta}^{(t)}) = d_{\boldsymbol{y}} \left(\sum_{\boldsymbol{z} \in \mathscr{Y}} d_{\boldsymbol{z}} \right)^{-1},$$

for $i \in \mathscr{P}_j$. Since $\tilde{\boldsymbol{Y}}$ is a discrete random vector, we could compute $P(\tilde{\boldsymbol{Y}}_i = \boldsymbol{y}|\boldsymbol{Z}, \boldsymbol{\delta}^{(t)})$ using the 'brute force' approach of summing $f(\tilde{\boldsymbol{Y}}|\boldsymbol{Z}, \boldsymbol{\delta}^{(t)})$ over all values of $\tilde{\boldsymbol{Y}}$ with $\tilde{\boldsymbol{Y}}_{\boldsymbol{i}} = \boldsymbol{y}$. However, this is computationally impractical. Instead, we subset $\tilde{\boldsymbol{Y}}$ into the components from individuals in pool j and the remaining components, that is, we take $\tilde{\boldsymbol{Y}} = (\tilde{\boldsymbol{Y}}_{l:l \in \mathscr{P}_j}, \tilde{\boldsymbol{Y}}_{l:l \notin \mathscr{P}_j})$. We then note that

$$f(\tilde{\boldsymbol{Y}}|\boldsymbol{Z}, \boldsymbol{\delta}^{(t)}) \propto f(\tilde{\boldsymbol{Y}}, \boldsymbol{Z}|\boldsymbol{\delta}^{(t)}) = f(\tilde{\boldsymbol{Y}}_{l:l \in \mathscr{P}_j}|\boldsymbol{Z}, \boldsymbol{\delta}^{(t)}) f(\boldsymbol{Y}_{l \notin \mathscr{P}_j}|\boldsymbol{Z}, \delta^{(t)})$$

where

$$f(\tilde{\boldsymbol{Y}}_{l:l\ \in\mathscr{P}_j}|\boldsymbol{Z},\boldsymbol{\delta}^{(t)}) = \prod_{k=1}^{K}\{Se_k^{\tilde{Z}_{jk}}(1-Sp_k)^{1-\tilde{Z}_{jk}}\}^{Z_{jk}}\{(1-Se_k)^{\tilde{Z}_{jk}}Sp_k^{1-\tilde{Z}_{jk}}\}^{1-Z_{jk}}$$
$$\times\prod_{l\in\mathscr{P}_j}\left[\left\{\prod_{\boldsymbol{z}\in\mathscr{Y}\setminus\boldsymbol{1}}\delta_{\boldsymbol{z}}^{(t)I(\tilde{\boldsymbol{Y}}_l=\boldsymbol{z})}\right\}\left(1-\sum_{\boldsymbol{z}\in\mathscr{Y}\setminus\boldsymbol{1}}\delta_{\boldsymbol{z}}^{(t)}\right)^{I(\tilde{\boldsymbol{Y}}_l=\boldsymbol{1})}\right]$$

and

$$f(\tilde{\boldsymbol{Y}}_{l:l\ \notin\mathscr{P}_j}|\boldsymbol{Z},\boldsymbol{\delta}^{(t)}) = \prod_{m=1,m\neq j}^{J}\prod_{k=1}^{K}\{Se_k^{\tilde{Z}_{mk}}(1-Sp_k)^{1-\tilde{Z}_{mk}}\}^{Z_{mk}}\{(1-Se_k)^{\tilde{Z}_{mk}}Sp_k^{1-\tilde{Z}_{mk}}\}^{1-Z_{mk}}$$
$$\times\prod_{l\in\mathscr{P}_m}\left[\left\{\prod_{\boldsymbol{z}\in\mathscr{Y}\setminus\boldsymbol{1}}\delta_{\boldsymbol{z}}^{(t)I(\tilde{\boldsymbol{Y}}_l=\boldsymbol{z})}\right\}\left(1-\sum_{\boldsymbol{z}\in\mathscr{Y}\setminus\boldsymbol{1}}\delta_{\boldsymbol{z}}^{(t)}\right)^{I(\tilde{\boldsymbol{Y}}_l=\boldsymbol{1})}\right].$$

Thus we have that $\tilde{\boldsymbol{Y}}_{l:l\in\mathscr{P}_j}$ is independent from $\tilde{\boldsymbol{Y}}_{l:l\notin\mathscr{P}_j}$ given $\boldsymbol{Z}$ and $\boldsymbol{\delta}^{(t)}$. Thus

$$P(\tilde{\boldsymbol{Y}}_{\boldsymbol{i}}=\boldsymbol{y}|\boldsymbol{Z},\boldsymbol{\delta}^{(t)}) \propto d_{\boldsymbol{y}} := \sum_{\tilde{\boldsymbol{Y}}_{l:l\in\mathscr{P}_j}\text{ with }\tilde{\boldsymbol{Y}}_{\boldsymbol{i}}=\boldsymbol{y}} f(\tilde{\boldsymbol{Y}}_{l:l\ \in\mathscr{P}_j}|\boldsymbol{Z},\boldsymbol{\delta}^{(t)})$$

where the proportionality constant is given by

$$\sum_{\tilde{\boldsymbol{Y}}_{l:l\in\mathscr{P}_j}} f(\tilde{\boldsymbol{Y}}_{l:l\ \in\mathscr{P}_j}|\boldsymbol{Z},\boldsymbol{\delta}^{(t)}) = \sum_{\boldsymbol{x}\in\mathscr{Y}} d_{\boldsymbol{x}}.$$

Note $d_{\boldsymbol{y}}$ depends on j only through $\boldsymbol{Z}_j$ and therefore $E[I(\tilde{\boldsymbol{Y}}_{\boldsymbol{i}}=\boldsymbol{y})|\boldsymbol{Z},\boldsymbol{\delta}^{(t)}]$ depends only on the observed value of $\boldsymbol{Z}_j$ and not directly on i or j. It is therefore only necessary to compute $E[I(\tilde{\boldsymbol{Y}}_{\boldsymbol{i}}=\boldsymbol{y})|\boldsymbol{Z},\boldsymbol{\delta}^{(t)}]$ once for each unique $\boldsymbol{Z}_j$ in the observed data set rather than once for each $i=1,...,N$. This reduces the required number of computations from N to (at most) 2^K.

Additional Details for Louis's Method

Recall that Louis's method defines

$$\widehat{I(\boldsymbol{\delta})} = -\frac{\partial^2 Q(\boldsymbol{\delta}, \hat{\boldsymbol{\delta}})}{\partial \boldsymbol{\delta}, \partial \boldsymbol{\delta}'} - \text{var}\left(\frac{\partial f(\boldsymbol{\delta}|\boldsymbol{Z}, \tilde{\boldsymbol{Y}})}{\partial \boldsymbol{\delta}} \middle| \hat{\boldsymbol{\delta}} \right).$$

Let $\boldsymbol{a}, \boldsymbol{b} \in \mathscr{Y}^* = \mathscr{Y} \setminus \mathbf{1}$ with $\boldsymbol{a} \neq \boldsymbol{b}$ and let $l(\boldsymbol{\delta}|\boldsymbol{Z}, \tilde{\boldsymbol{Y}}) = \log\{f(\boldsymbol{\delta}|\boldsymbol{Z}, \tilde{\boldsymbol{Y}})\}$. We have that

$$\frac{\partial^2 l}{\partial \delta_{\boldsymbol{a}}^2} = \sum_{j=1}^{J} \sum_{i \in \mathscr{P}_j} -I(\tilde{\boldsymbol{Y}}_{\boldsymbol{i}} = \boldsymbol{a})\delta_{\boldsymbol{a}}^{-2} - I(\tilde{\boldsymbol{Y}}_{\boldsymbol{i}} = \mathbf{1})(1 - \sum_{\boldsymbol{y} \in \mathscr{Y}^*} \delta_{\boldsymbol{y}})^{-2}$$
$$\frac{\partial^2 l}{\partial \delta_{\boldsymbol{a}} \partial \delta_{\boldsymbol{b}}} = \sum_{j=1}^{J} \sum_{i \in \mathscr{P}_j} -I(\tilde{\boldsymbol{Y}}_{\boldsymbol{i}} = \mathbf{1})(1 - \sum_{\boldsymbol{y} \in \mathscr{Y}^*} \delta_{\boldsymbol{y}})^{-2}$$

Therefore

$$\frac{\partial^2 Q(\boldsymbol{\delta}, \hat{\boldsymbol{\delta}})}{\partial \delta_{\boldsymbol{a}}^2} = \sum_{j=1}^{J} \sum_{i \in \mathscr{P}_j} -E(\tilde{\boldsymbol{Y}}_{\boldsymbol{i}} = \boldsymbol{a})\hat{\delta}_{\boldsymbol{a}}^{-2} - E(\tilde{\boldsymbol{Y}}_{\boldsymbol{i}} = \mathbf{1})(1 - \sum_{\boldsymbol{y} \in \mathscr{Y}^*} \hat{\delta}_{\boldsymbol{y}})^{-2}$$
$$\frac{\partial^2 Q(\boldsymbol{\delta}, \hat{\boldsymbol{\delta}})}{\partial \delta_{\boldsymbol{a}} \partial \delta_{\boldsymbol{b}}} = \sum_{j=1}^{J} \sum_{i \in \mathscr{P}_j} -E(\tilde{\boldsymbol{Y}}_{\boldsymbol{i}} = \mathbf{1})(1 - \sum_{\boldsymbol{y} \in \mathscr{Y}^*} \hat{\delta}_{\boldsymbol{y}})^{-2}.$$

Note that the expectations are taken conditional on the observed data $\boldsymbol{Z}$. However, since pool responses in different pools are independent, we have $E(\tilde{\boldsymbol{Y}}_{\boldsymbol{i}} = \boldsymbol{a}) = E(\tilde{\boldsymbol{Y}}_{\boldsymbol{k}} = \boldsymbol{a})$ for all $i \in \mathscr{P}_j$ and all $k \in \mathscr{P}_l$ for which $\boldsymbol{Z}_j = \boldsymbol{Z}_l$. That is, the expectations depend only the observed pool response and not on the individual i or the specific pool j. This is computationally convenient, as it reduces the number of expectations which must be computed for each $\boldsymbol{a}$ from N to (at most) 2^K. We also note that these expectations are computed as part of the EM algorithm and thus the expectation computed on the final iteration of the algorithm may be used here.

Note that

$$\frac{\partial l}{\partial \delta_{\boldsymbol{a}}} = \sum_{j=1}^{J} \sum_{i \in \mathscr{P}_j} I(\tilde{\boldsymbol{Y}}_{\boldsymbol{i}} = \boldsymbol{a})\delta_{\boldsymbol{a}}^{-1} - I(\tilde{\boldsymbol{Y}}_{\boldsymbol{i}} = \boldsymbol{y}^*)(1 - \sum_{\boldsymbol{y} \in \mathscr{Y}^*} \delta_{\boldsymbol{y}})^{-1}$$

And thus

$$\begin{aligned}\operatorname{var}\left(\frac{\partial l}{\partial \delta_{\boldsymbol{a}}}\bigg|\hat{\boldsymbol{\delta}}\right) &= \sum_{j=1}^{J} \operatorname{var}\left[\sum_{i\in\mathscr{P}_j} I(\tilde{\boldsymbol{Y}}_{\boldsymbol{i}} = \boldsymbol{a})\hat{\delta}_{\boldsymbol{a}}^{-1} - I(\tilde{\boldsymbol{Y}}_{\boldsymbol{i}} = \boldsymbol{1})(1 - \sum_{\boldsymbol{y}\in\mathscr{Y}^*} \hat{\delta}_{\boldsymbol{y}})^{-1}\right] \\ &= \sum_{j=1}^{J} E\left\{\left[\sum_{i\in\mathscr{P}_j} I(\tilde{\boldsymbol{Y}}_{\boldsymbol{i}} = \boldsymbol{a})\hat{\delta}_{\boldsymbol{a}}^{-1} - I(\tilde{\boldsymbol{Y}}_{\boldsymbol{i}} = \boldsymbol{1})(1 - \sum_{\boldsymbol{y}\in\mathscr{Y}^*} \hat{\delta}_{\boldsymbol{y}})^{-1}\right]^2\right\} \\ &- \left\{E\left[\sum_{i\in\mathscr{P}_j} I(\tilde{\boldsymbol{Y}}_{\boldsymbol{i}} = \boldsymbol{a})\hat{\delta}_{\boldsymbol{a}}^{-1} - I(\tilde{\boldsymbol{Y}}_{\boldsymbol{i}} = \boldsymbol{1})(1 - \sum_{\boldsymbol{y}\in\mathscr{Y}^*} \hat{\delta}_{\boldsymbol{y}})^{-1}\right]\right\}^2\end{aligned}$$

Note that these expectations again depend only on the observed value $\boldsymbol{Z}_j$ and not directly on j or i, and thus we need only compute 2^K expectations for each $\boldsymbol{a}$. Once again, partition $\tilde{\boldsymbol{Y}} = (\tilde{\boldsymbol{Y}}_{l:l\in\mathscr{P}_j}, \tilde{\boldsymbol{Y}}_{l:l\notin\mathscr{P}_j})$ and let $\boldsymbol{y}_{l:l\in\mathscr{P}_j} = (\boldsymbol{y}_1, \boldsymbol{y}_2, ..., \boldsymbol{y}_c)'$ for $\boldsymbol{y}_i \in \mathscr{Y}$. We have that

$$P(\tilde{\boldsymbol{Y}}_{l:l\in\mathscr{P}_j} = \boldsymbol{y}_{l:l\in\mathscr{P}_j}|\boldsymbol{Z}_j, \hat{\boldsymbol{\delta}}) = \frac{p_{\boldsymbol{y}_{l:l\in\mathscr{P}_j}}}{\sum_{\boldsymbol{x}\in\mathscr{Y}^c} p_{\boldsymbol{x}}}$$

where $\mathscr{Y}^c$ denotes the set product of $\mathscr{Y}$ with itself c times and

$$\begin{aligned}p_{\boldsymbol{y}_{l:l\in\mathscr{P}_j}} &= \prod_{k=1}^{K}\{Se_k^{\tilde{z}_k}(1-Sp_k)^{1-\tilde{z}_k}\}^{Z_{jk}}\{(1-Se_k)^{\tilde{z}_k}Sp_k^{1-\tilde{z}_k}\}^{1-Z_{jk}} \\ &\times \prod_{l=1}^{c}\left\{\left[\prod_{\boldsymbol{z}\in\mathscr{Y}^*} \hat{\delta}_{\boldsymbol{z}}^{I(\boldsymbol{y}_l=\boldsymbol{z})}\right]\left(1 - \sum_{\boldsymbol{z}\in\mathscr{Y}^*} \hat{\delta}_{\boldsymbol{z}}\right)^{I(\boldsymbol{y}_l=\boldsymbol{1})}\right\}\end{aligned}$$

and $\tilde{\boldsymbol{z}}$ is the pool status corresponding to $\boldsymbol{y}_{l:l\in\mathscr{P}_j}$. Thus we can compute the expectations in (2) and (3) by noting that they are a function of $\boldsymbol{Y}_{l:l\in\mathscr{P}_j}$ and using $P(\boldsymbol{Y}_{l:l\in\mathscr{P}_j}|\boldsymbol{Z}_j, \hat{\boldsymbol{\delta}})$.

For $\boldsymbol{a} \neq \boldsymbol{b}$, we have that

$$\operatorname{cov}\left(\frac{\partial l}{\partial \delta_{\boldsymbol{a}}}\bigg|\hat{\boldsymbol{\delta}}, \frac{\partial l}{\partial \delta_{\boldsymbol{b}}}\bigg|\hat{\boldsymbol{\delta}}\right)$$

$$= \sum_{j=1}^{J} \text{cov} \left(\sum_{i \in \mathscr{P}_j} I(\tilde{\boldsymbol{Y}}_{\boldsymbol{i}} = \boldsymbol{a})\delta_{\boldsymbol{a}}^{-1} - I(\tilde{\boldsymbol{Y}}_{\boldsymbol{i}} = \boldsymbol{y}^*)(1 - \sum_{\boldsymbol{y} \in \mathscr{Y}^*} \delta_{\boldsymbol{y}})^{-1}, \right.$$

$$\left. \sum_{i \in \mathscr{P}_j} I(\tilde{\boldsymbol{Y}}_{\boldsymbol{i}} = \boldsymbol{b})\delta_{\boldsymbol{b}}^{-1} - I(\tilde{\boldsymbol{Y}}_{\boldsymbol{i}} = \boldsymbol{y}^*)(1 - \sum_{\boldsymbol{y} \in \mathscr{Y}^*} \delta_{\boldsymbol{y}})^{-1} \right)$$

$$= \sum_{j=1}^{J} E \left\{ \left(\sum_{i \in \mathscr{P}_j} I(\tilde{\boldsymbol{Y}}_{\boldsymbol{i}} = \boldsymbol{a})\delta_{\boldsymbol{a}}^{-1} - I(\tilde{\boldsymbol{Y}}_{\boldsymbol{i}} = \boldsymbol{y} \setminus \boldsymbol{1})(1 - \sum_{\boldsymbol{y} \in \mathscr{Y}^*} \delta_{\boldsymbol{y}})^{-1} \right) \right.$$

$$\left. \times \left(\sum_{i \in \mathscr{P}_j} I(\tilde{\boldsymbol{Y}}_{\boldsymbol{i}} = \boldsymbol{b})\delta_{\boldsymbol{b}}^{-1} - I(\tilde{\boldsymbol{Y}}_{\boldsymbol{i}} = \boldsymbol{y}^*)(1 - \sum_{\boldsymbol{y} \in \mathscr{Y}^*} \delta_{\boldsymbol{y}})^{-1} \right) \right\}$$

$$- \left\{ E \left(\sum_{i \in \mathscr{P}_j} I(\tilde{\boldsymbol{Y}}_{\boldsymbol{i}} = \boldsymbol{a})\delta_{\boldsymbol{a}}^{-1} - I(\tilde{\boldsymbol{Y}}_{\boldsymbol{i}} = \boldsymbol{y}^*)(1 - \sum_{\boldsymbol{y} \in \mathscr{Y}^*} \delta_{\boldsymbol{y}})^{-1} \right) \right.$$

$$\left. \times E \left(\sum_{i \in \mathscr{P}_j} I(\tilde{\boldsymbol{Y}}_{\boldsymbol{i}} = \boldsymbol{b})\delta_{\boldsymbol{b}}^{-1} - I(\tilde{\boldsymbol{Y}}_{\boldsymbol{i}} = \boldsymbol{y}^*)(1 - \sum_{\boldsymbol{y} \in \mathscr{Y}^*} \delta_{\boldsymbol{y}})^{-1} \right) \right\}.$$

Again, these expectations depend only on the value of $\boldsymbol{Z}_j$ and not directly on j or i and thus we need only compute 2^K expectations for each pair of $\boldsymbol{a}$ and $\boldsymbol{b}$. Note that we can compute these expectations using the same $P(\tilde{\boldsymbol{Y}}_{\mathscr{P}_j} | \boldsymbol{Z}_j, \hat{\boldsymbol{\delta}})$ used to compute $\text{var}(\frac{\partial l}{\partial \boldsymbol{a}})$.

Additional Details for the Independent Model

In order to fit the model which assumes independent co-infections from Section 2.3, we must compute

$$E\left[I(\tilde{Y}_{ik} = l | \boldsymbol{Z}, \boldsymbol{\pi}^{(t)}) \right] = P(\tilde{Y}_{ik} = l | \boldsymbol{Z}, \boldsymbol{\pi}^{(t)})$$

for $i \in \mathscr{P}_j$ and $l \in \{0, 1\}$. Just as in the full multinomial model, we can show $\tilde{\boldsymbol{Y}}_{l:l \in \mathscr{P}_j}$ and $\tilde{\boldsymbol{Y}}_{l:l \notin \mathscr{P}_j}$ are independent given $\boldsymbol{Z}$ and $\boldsymbol{\pi}$. Take

$$f(\tilde{\boldsymbol{Y}}_{l:l\ \in \mathscr{P}_j} | \boldsymbol{Z}, \boldsymbol{\delta}^{(t)}) = \prod_{k=1}^{K} \{ Se_k^{\tilde{Z}_{jk}} (1 - Sp_k)^{1 - \tilde{Z}_{jk}} \}^{Z_{jk}} \{ (1 - Se_k)^{\tilde{Z}_{jk}} Sp_k^{1 - \tilde{Z}_{jk}} \}^{1 - Z_{jk}}$$
$$\times \prod_{l \in \mathscr{P}_j} \prod_{\boldsymbol{z} \in \mathscr{Y}} \delta_{\boldsymbol{z}}^{(t) I(\tilde{\boldsymbol{Y}}_{\boldsymbol{l}} = \boldsymbol{z})},$$

where $\boldsymbol{\delta}_{\boldsymbol{z}} = \prod_{k=1}^{K} \pi_k^{z_k}(1-\pi_k)^{1-z_k}$. Therefor

$$P(\tilde{Y}_{ik} = l|\boldsymbol{Z}, \boldsymbol{\pi}^{(t)}) \propto c_{kl} := \sum_{\tilde{\boldsymbol{Y}}_{l:l \in \mathscr{P}_j} \text{ with } \tilde{Y}_{ik}=l} f(\tilde{\boldsymbol{Y}}_{l:l\ \in \mathscr{P}_j}|\boldsymbol{Z}, \boldsymbol{\delta}^{(t)})$$

with proportionality constant $c_{k0} + c_{k1}$.

Web Appendix B

This section provides additional details about the relationship between individual-level and pool-level infection probabilities.

Given a pool size c and individual infection probabilities $\boldsymbol{\delta}$, we may wish to calculate $\theta_{\boldsymbol{z}} = P(\tilde{Z}_j = \boldsymbol{z}|\boldsymbol{\delta})$, the probability that a pool of size c has true infection status $\boldsymbol{z}$ given $\boldsymbol{\delta}$. We first note that $\theta_{\boldsymbol{0}} = \delta_{\boldsymbol{0}}^{c}$. For $1 \leq k \leq K$, define $\mu_{\boldsymbol{z}} = P(\tilde{Z}_{jk} = 0 \forall k : z_k = 0)$, that is $\mu_{\boldsymbol{z}}$ is the probability that a pool of size c contains no individuals infected with any pathogens k for which $\boldsymbol{z}_k = 0$. We have that $\mu_{\boldsymbol{z}} = (\sum_{\boldsymbol{x}:x_k=0\forall k:z_k=0} \delta_{\boldsymbol{x}})^c$. We also have that $\mu_{\boldsymbol{z}} = \sum_{\boldsymbol{x}:x_k=0\forall k:z_k=0} \theta_{\boldsymbol{x}}$. This produces a system of 2^k non-linear equations in 2^k unknowns. The system can be easily solved for $\boldsymbol{\theta} = (\theta_{\boldsymbol{z}})_{\boldsymbol{z}\in\mathscr{Y}}$ by starting with $\theta_{\boldsymbol{0}} = \boldsymbol{\delta}_0^c$. The equations derived from $\mu_{\boldsymbol{z}}$ for $\boldsymbol{z}$ with exactly one entry equal to 1 each involve only $\theta_{\boldsymbol{0}}$ and $\theta_{\boldsymbol{z}}$, which allows us to next solve for $\theta_{\boldsymbol{z}}$ for all $\boldsymbol{z}$ with exactly one 1. Each equation involving $\mu_{\boldsymbol{z}}$ with exactly two entries equal to 1 each involve only $\theta_{\boldsymbol{x}}$s previously solved for and $\theta_{\boldsymbol{z}}$. This pattern continues, allowing us to solve for all components of $\boldsymbol{\theta}$.

Web Appendix C

Web Figures 1-6 provide enlarged versions of the panels from Figure 1; Web Figures 7-12 provide enlarged versions of the panes from Figure 2.

We conducted a simulation study to assess the performance of the test for mutual independence described in Section 2.3. We generated data using $c = 3$ for each of the $\boldsymbol{\delta}$ values in Table 1, with $Se_k = Sp_k = 0.99$ for all k. Supplementary Table 1 provides the percent

Scenario	1	2	3	4	5	6
Rejection Rate	7%	100%	100%	10%	100%	100%

Web Table 1: The table displays the empirical rejection rate of the null hypothesis of independence using data generated with each value of $\boldsymbol{\delta}$ shown in Table 1 and $c = 3$.

of datasets for which the likelihood ratio test statistic exceeded the 95th quantile of a chi-squared distribution with 4 degrees of freedom. For the independent scenarios (1 and 4), this quantity is the empirical type I error rate. For the other scenarios, this quantity is the empirical power.

Web Appendix D

This section provides additional details about the likelihood ratio test used in Section 5.

Recall that we wish to test $H_0 : \delta_{1100} = \delta_{1010} = \delta_{1110} = \delta_{0101} = \delta_{1101} = \delta_{0011} = \delta_{1011} = \delta_{0111} = \delta_{1111}$ versus $H_A : \delta_{1100}, \delta_{1010}, \delta_{1110}, \delta_{0101}, \delta_{1101}, \delta_{0011}, \delta_{1011}, \delta_{0111}, \delta_{1111}$ not all 0. Note that since under H_A, $0 \leq \delta_{\boldsymbol{z}}$ for all $\boldsymbol{z}$, the null hypothesis exists on the boundary of the parameter space for the alternative hypothesis, and thus the assumptions of the usual likelihood ratio test are violated. As such, we have no guarantee that the null distribution of the likelihood ratio test statistic follows the usual chi-squared distribution. To circumvent this, we use a Monte Carlo procedure to estimate the null distribution of the test statistic. We generated 100 datasets by independently generating $n = 573$ individual infection statuses from a multinomial distribution with $\boldsymbol{\delta} = \hat{\boldsymbol{\delta}}$ (Reduced) from Table 2. We randomly assigned these individuals to 191 pools of size 3 and generated pool testing responses as described in Section 3. We then fit both the full and reduced model to each datasets and computed the likelihood ratio test statistics. We then took the 95th quantile of the sample of 100 test statistics as the critical value for our hypothesis test.

Web Appendix E

We performed a sensitivity analysis using the data described in Section 4 taking $Se_k = Sp_k = 0.97$. When performing the test for independence, we found a likelihood ratio test statistic

	R. amblyommatis	*R. parkeri*	*PME*	*E. ewingii*	$\hat{\boldsymbol{\delta}}$ (Full)	$\hat{\boldsymbol{\delta}}$ (Reduced)
	0	0	0	0	0.6162	0.6162
	1	0	0	0	0.3153	0.3153
	0	1	0	0	0.0615	0.0615
	1	1	0	0	$<10^{-50}$	0
	0	0	1	0	0.0014	0.0014
	1	0	1	0	$<10^{-50}$	0
	0	1	1	0	0.0016	0.0016
	1	1	1	0	$<10^{-50}$	0
	0	0	0	1	9.6119×10^{-21}	7.8518×10^{-21}
	1	0	0	1	0.0042	0.0042
	0	1	0	1	$<10^{-50}$	0
	1	1	0	1	$<10^{-50}$	0
	0	0	1	1	$<10^{-50}$	0
	1	0	1	1	$<10^{-50}$	0
	0	1	1	1	$<10^{-50}$	0
	1	1	1	1	$<10^{-50}$	0
$\hat{p}$ (Full)	0.3194	0.0630	0.0029	0.0042		
$\hat{p}$ (Reduced)	0.3194	0.0630	0.0029	0.0042		

Web Table 2: The table provides the estimated multinomial probabilities ($\hat{\boldsymbol{\delta}}$) and estimated marginal prevalences ($\hat{\boldsymbol{p}}$) for the South Carolina tick data.

of $17.64 > \chi_{0.95,9} = 16.92$, so we rejected the null hypothesis of independence. The estimate of $\hat{\boldsymbol{\delta}}$ from the full multinomial model is provided in Web Table 2.

We refit the model setting $\boldsymbol{\delta}_{\boldsymbol{y}} = 0$ for all $\boldsymbol{y}$ for which $\hat{\boldsymbol{\delta}}_{\boldsymbol{y}} < 10^{-50}$. We used a likelihood ratio test to compare this reduced model to the full model and failed to reject the null hypothesis. We report $\hat{\boldsymbol{\delta}}$ from the reduced model in Table 2.

As in the original data application, we applied the delta method to examine *R. parkeri*-PME and *R. amblyommatis*-*E. ewingii* co-infections using the reduced model. We found $\hat{d}(2,3) = -0.0012$, 95% confidence interval $(-0.0013, -0.0012)$ and thus conclude that co-infection with *R. parkeri* and PME is more likely than expected under independence. We also found $\hat{d}(1,4) = -0.0046$, 95% confidence interval $(-0.0047, -0.0046)$ and conclude that infection with *R. amblyommatis* and *E. ewingii* are positively associated. These results are consistent with those of the primary analysis.

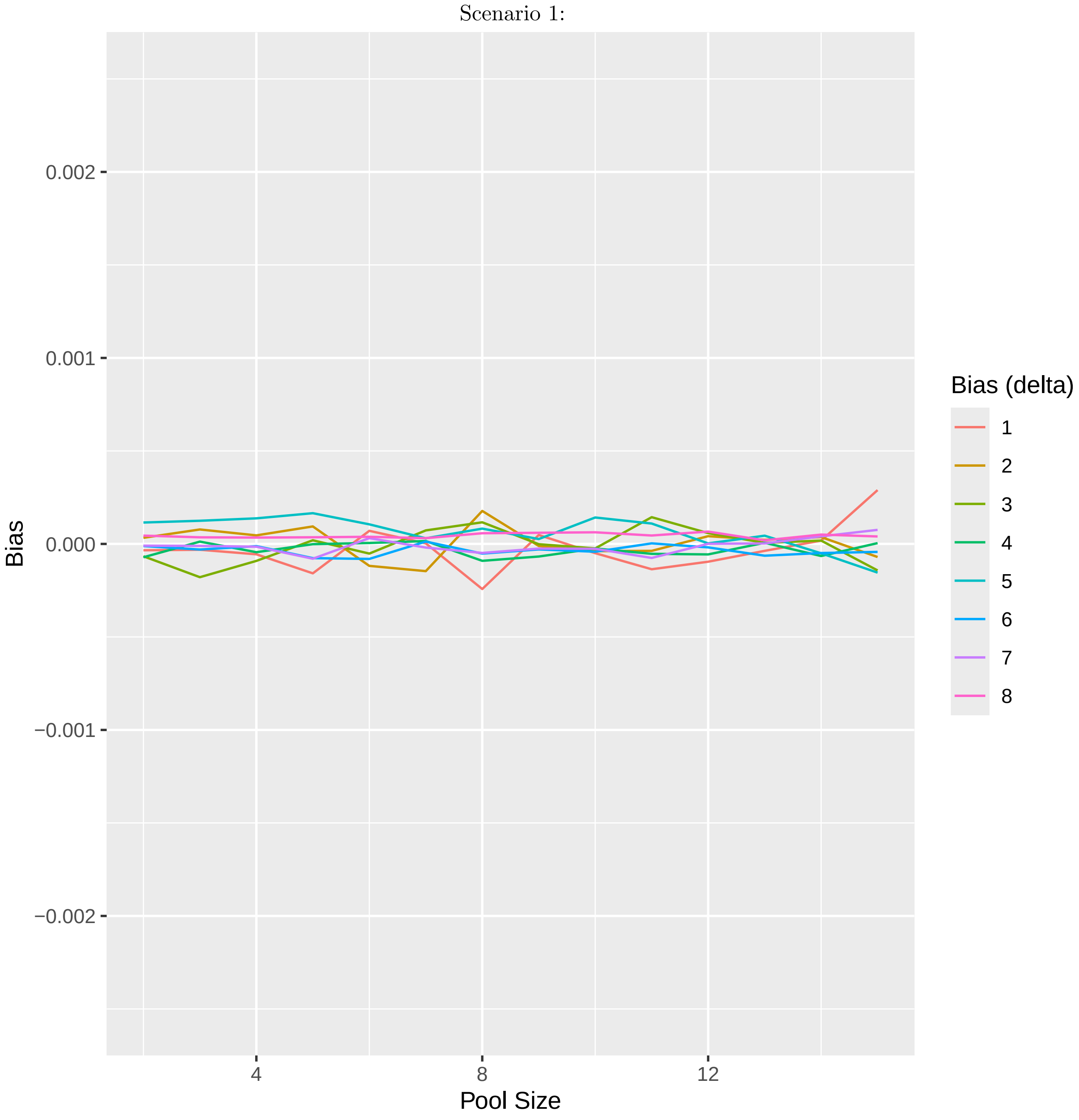

Web Fig. 1: The figure displays the average empirical bias of the estimator $\hat{\boldsymbol{\delta}}$ from scenario 1 as a function of pool size. From 1 to 8, the position numbers correspond to $\boldsymbol{y} = (0,0,0)$, $(1,0,0)$, $(0,1,0)$, $(1,0,1)$, $(1,1,0)$, $(0,1,1)$ and $(1,1,1)$. This is an enlarged version of Figure 1(a).

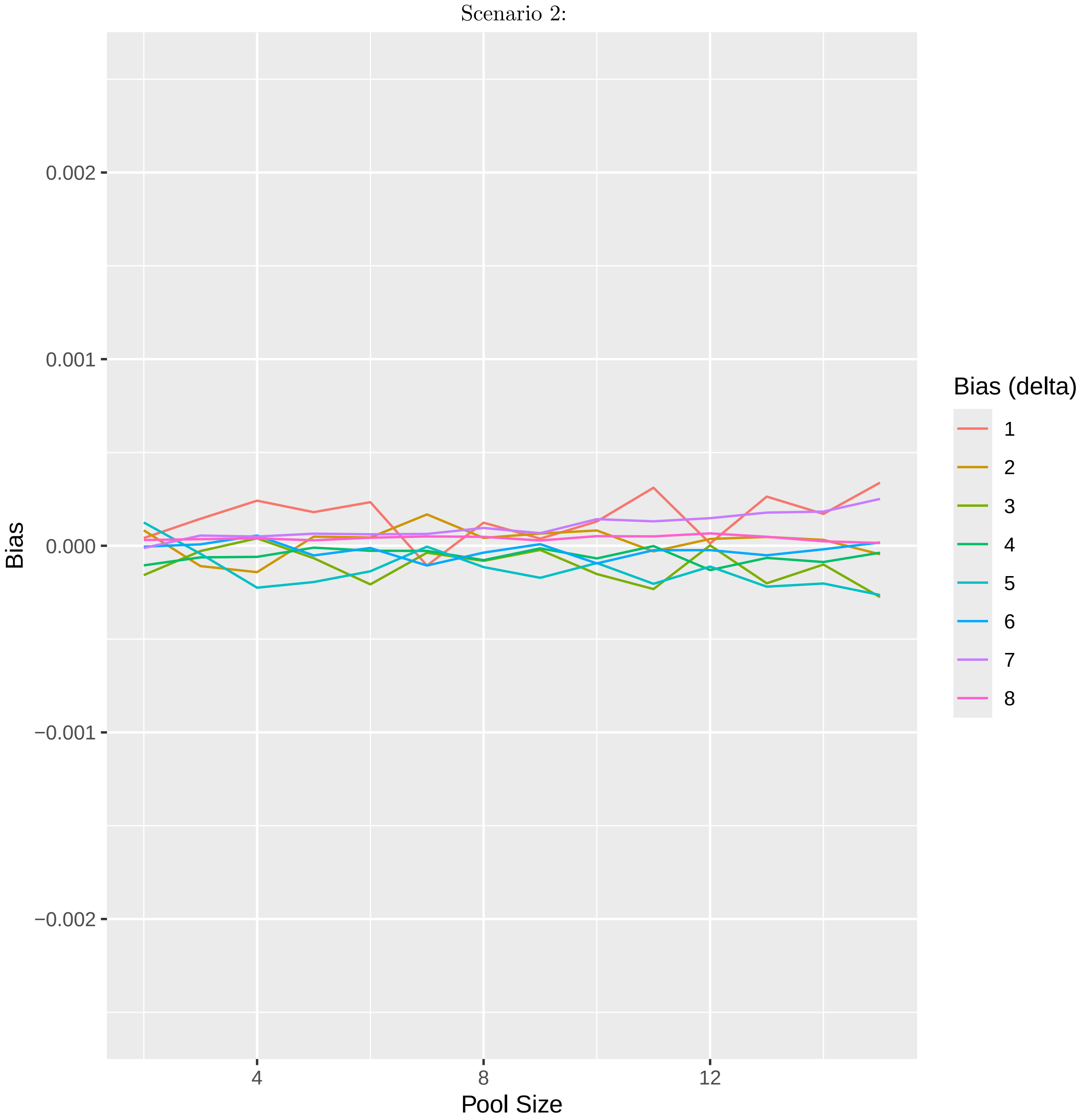

Web Fig. 2: The figure displays the average empirical bias of the estimator $\hat{\boldsymbol{\delta}}$ from scenario 2 as a function of pool size. From 1 to 8, the position numbers correspond to $\boldsymbol{y} = (0, 0, 0)$, $(1, 0, 0)$, $(0, 1, 0)$, $(1, 0, 1)$, $(1, 1, 0)$, $(0, 1, 1)$ and $(1, 1, 1)$. This is an enlarged version of Figure 1(b).

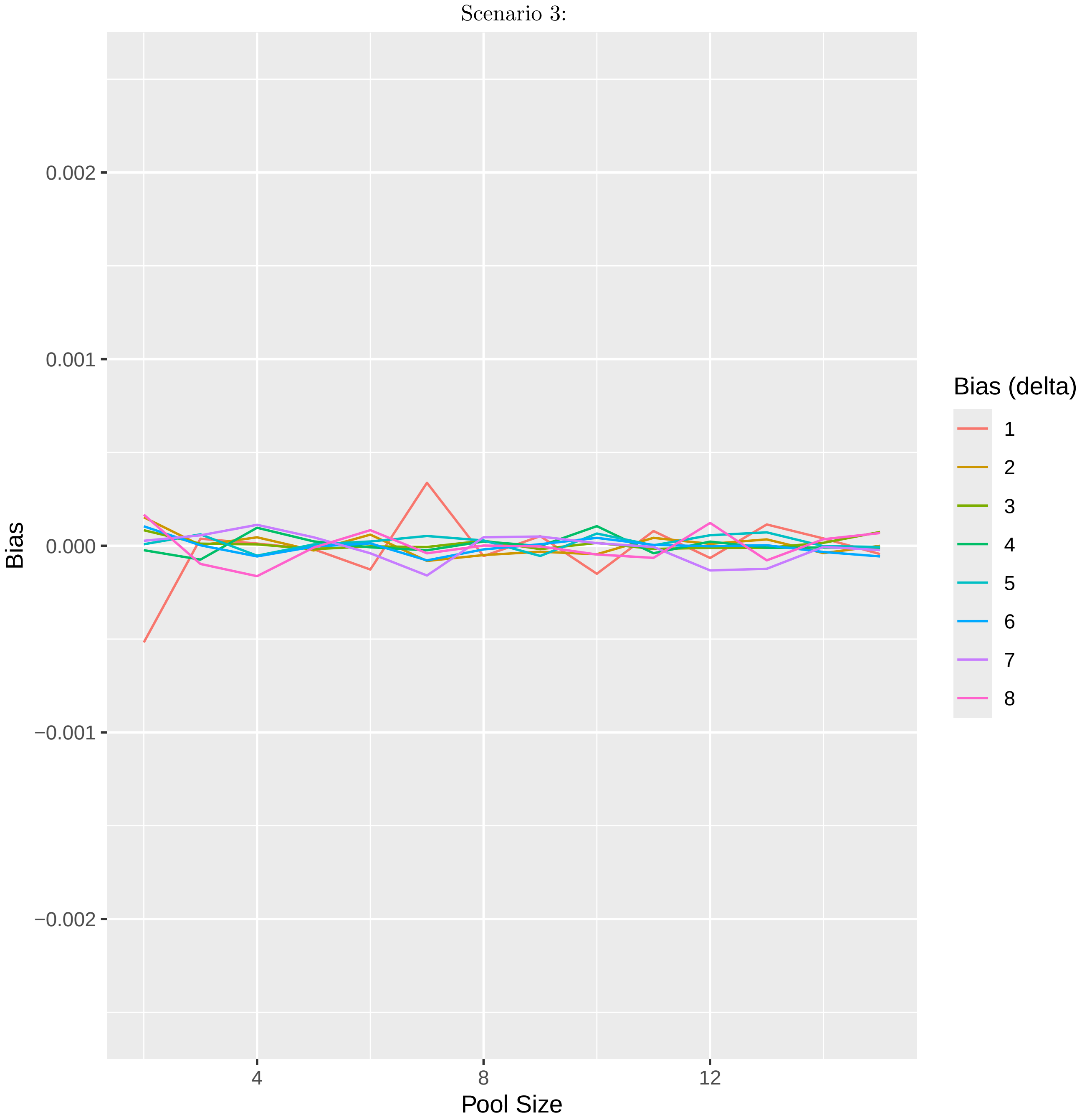

Web Fig. 3: The figure displays the average empirical bias of the estimator $\hat{\boldsymbol{\delta}}$ from scenario 3 as a function of pool size. From 1 to 8, the position numbers correspond to $\boldsymbol{y} = (0,0,0)$, $(1,0,0)$, $(0,1,0)$, $(1,0,1)$, $(1,1,0)$, $(0,1,1)$ and $(1,1,1)$. This is an enlarged version of Figure 1(c).

Scenario 4:

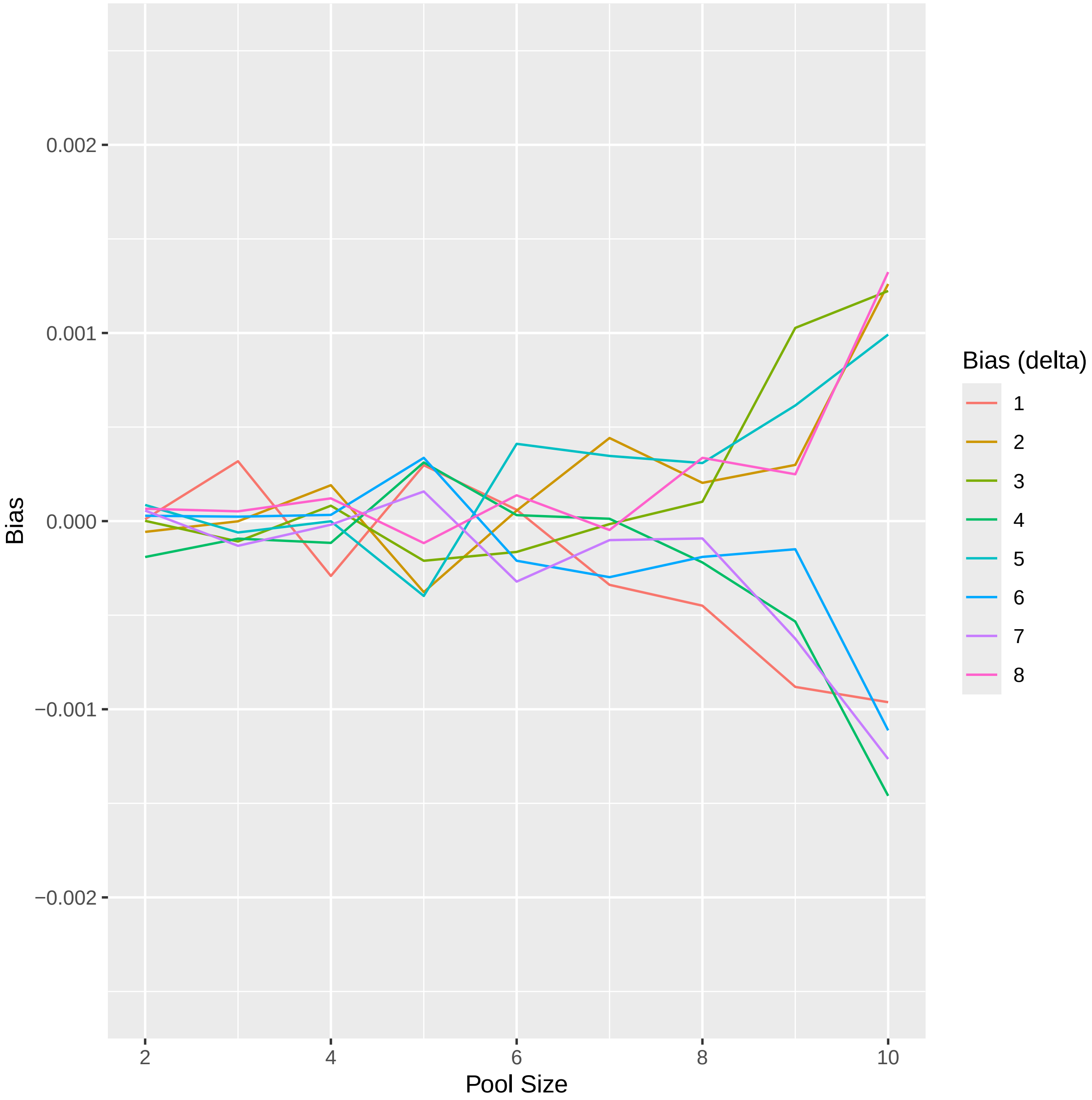

Web Fig. 4: The figure displays the average empirical bias of the estimator $\hat{\boldsymbol{\delta}}$ from scenario 4 as a function of pool size. From 1 to 8, the position numbers correspond to $\boldsymbol{y} = (0, 0, 0)$, $(1, 0, 0)$, $(0, 1, 0)$, $(1, 0, 1)$, $(1, 1, 0)$, $(0, 1, 1)$ and $(1, 1, 1)$. This is an enlarged version of Figure 1(d).

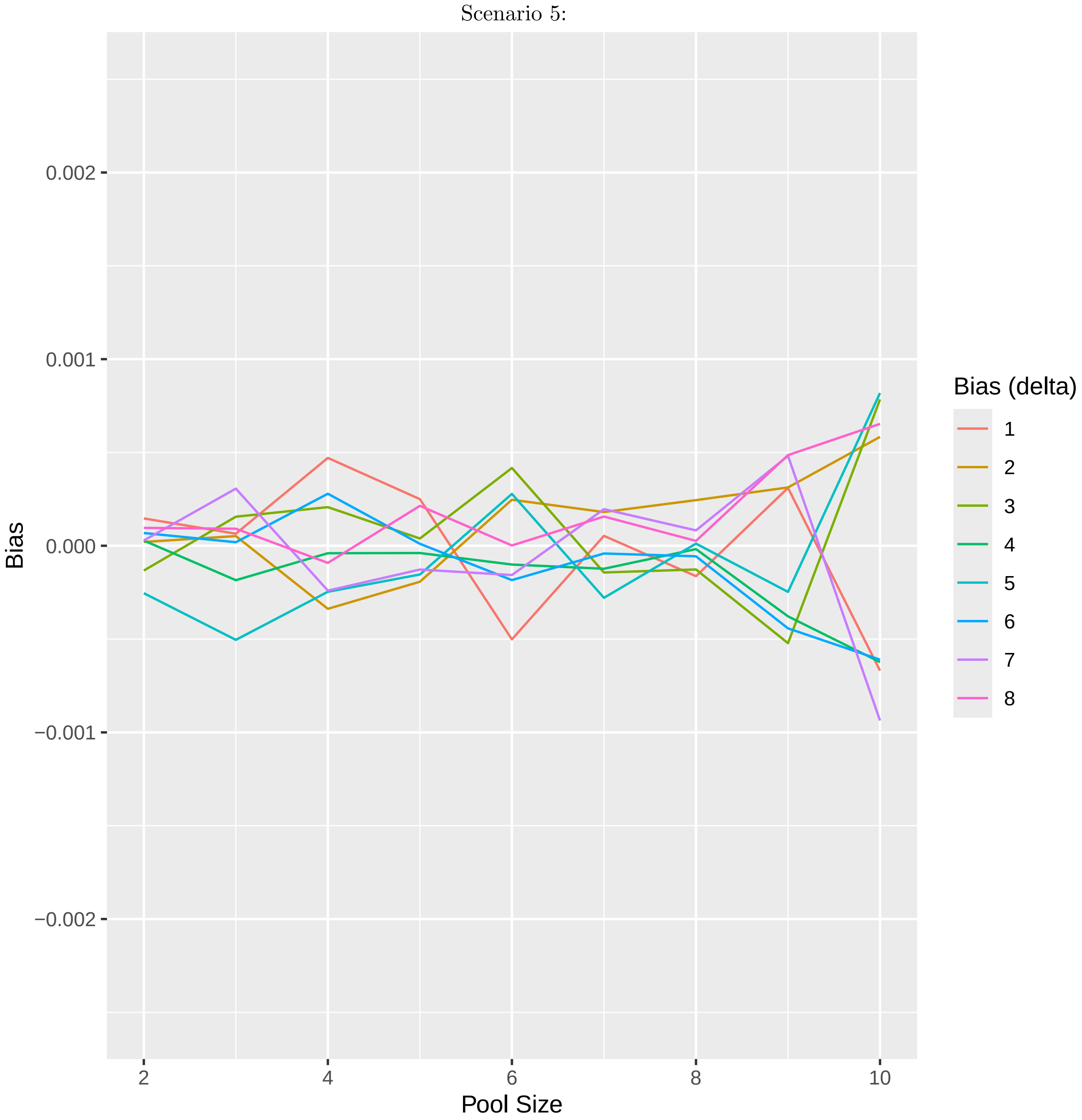

Web Fig. 5: The figure displays the average empirical bias of the estimator $\hat{\boldsymbol{\delta}}$ from scenario 5 as a function of pool size. From 1 to 8, the position numbers correspond to $\boldsymbol{y} = (0,0,0)$, $(1,0,0)$, $(0,1,0)$, $(1,0,1)$, $(1,1,0)$, $(0,1,1)$ and $(1,1,1)$. This is an enlarged version of Figure 1(e).

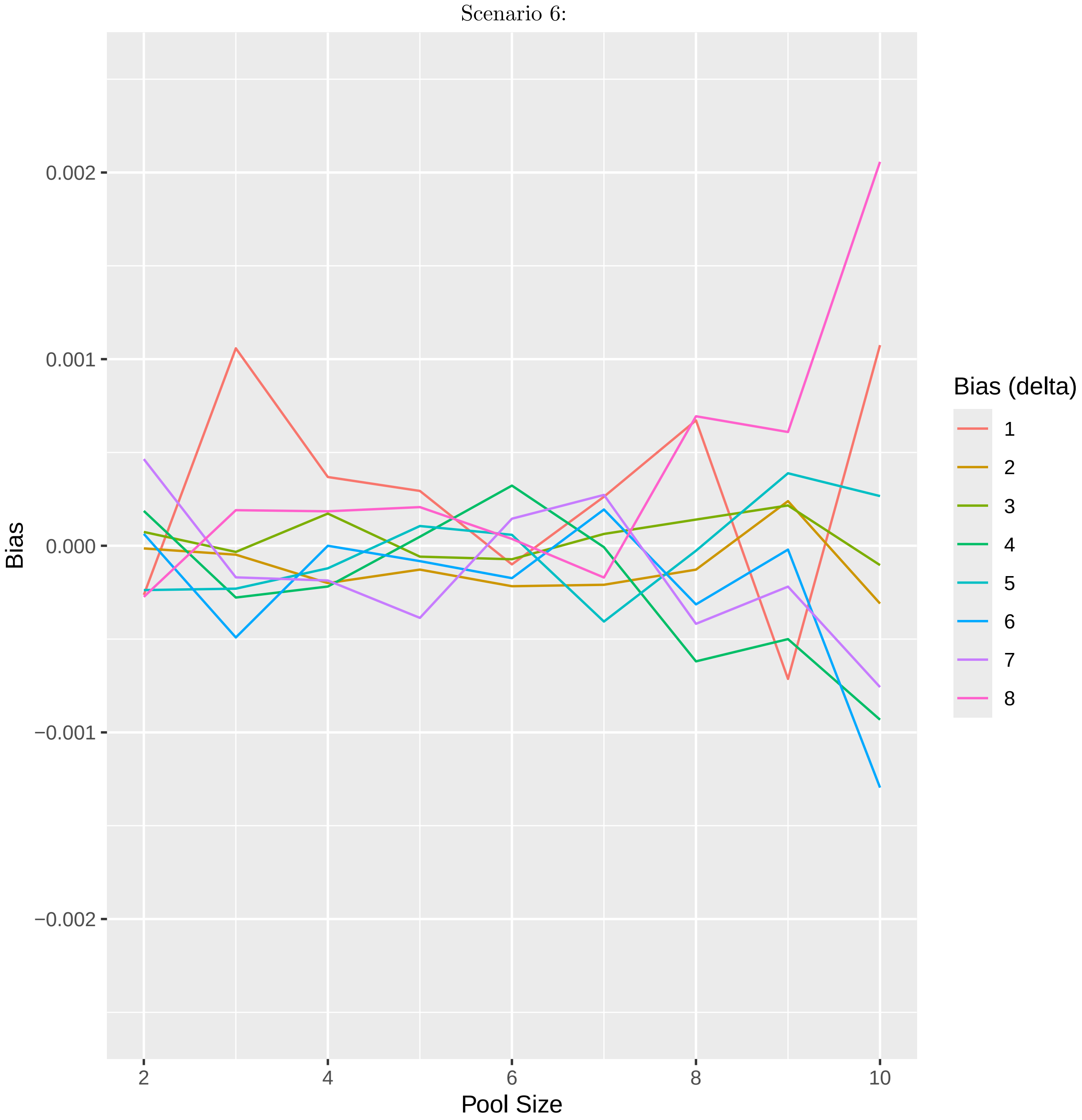

Web Fig. 6: The figure displays the average empirical bias of the estimator $\hat{\boldsymbol{\delta}}$ from scenario 6 as a function of pool size. From 1 to 8, the position numbers correspond to $\boldsymbol{y} = (0,0,0)$, $(1,0,0)$, $(0,1,0)$, $(1,0,1)$, $(1,1,0)$, $(0,1,1)$ and $(1,1,1)$. This is an enlarged version of Figure 1(f).

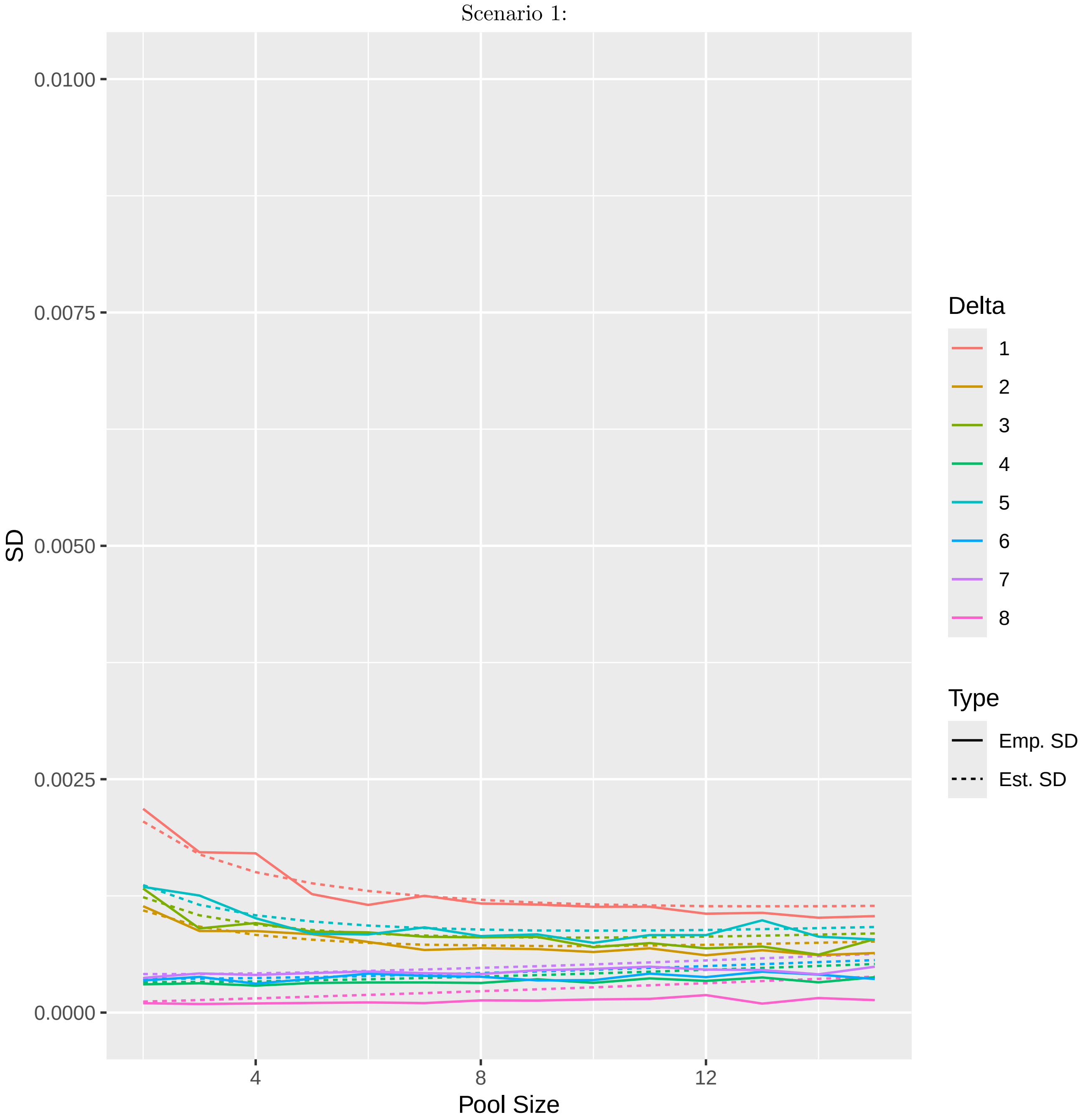

Web Fig. 7: The figure displays the average estimated standard error from Louis's method and the standard deviation of the sample of point estimators for $\hat{\boldsymbol{\delta}}$ from scenario 1 as a function of pool size. From 1 to 8, the position numbers correspond to $\boldsymbol{y} = (0,0,0)$, $(1,0,0)$, $(0,1,0)$, $(1,0,1)$, $(1,1,0)$, $(0,1,1)$ and $(1,1,1)$. This is an enlarged version of Figure 2(a).

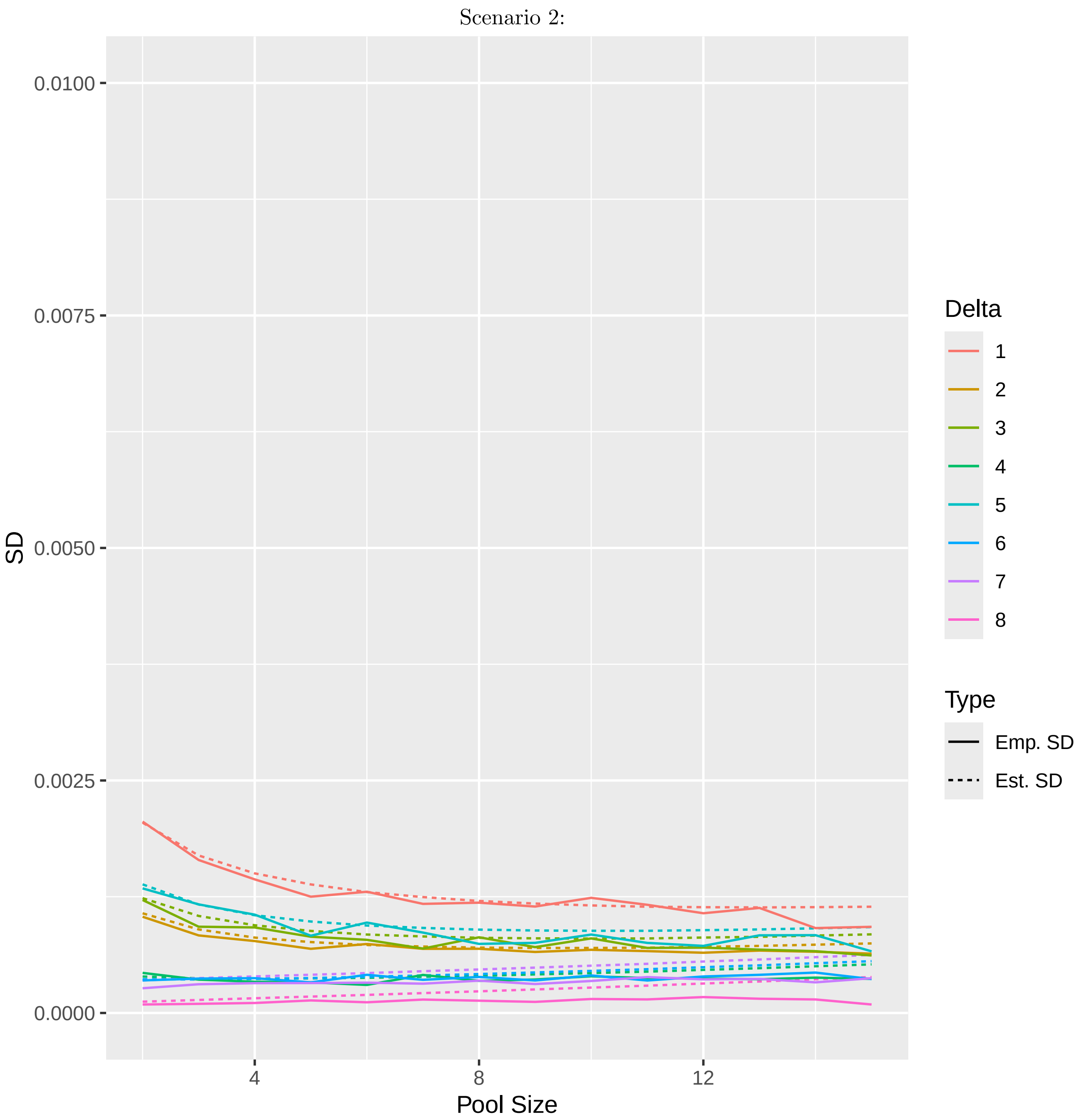

Web Fig. 8: The figure displays the average estimated standard error from Louis's method and the standard deviation of the sample of point estimators for $\hat{\boldsymbol{\delta}}$ from scenario 2 as a function of pool size. From 1 to 8, the position numbers correspond to $\boldsymbol{y} = (0, 0, 0)$, $(1, 0, 0)$, $(0, 1, 0)$, $(1, 0, 1)$, $(1, 1, 0)$, $(0, 1, 1)$ and $(1, 1, 1)$. This is an enlarged version of Figure 2(b).

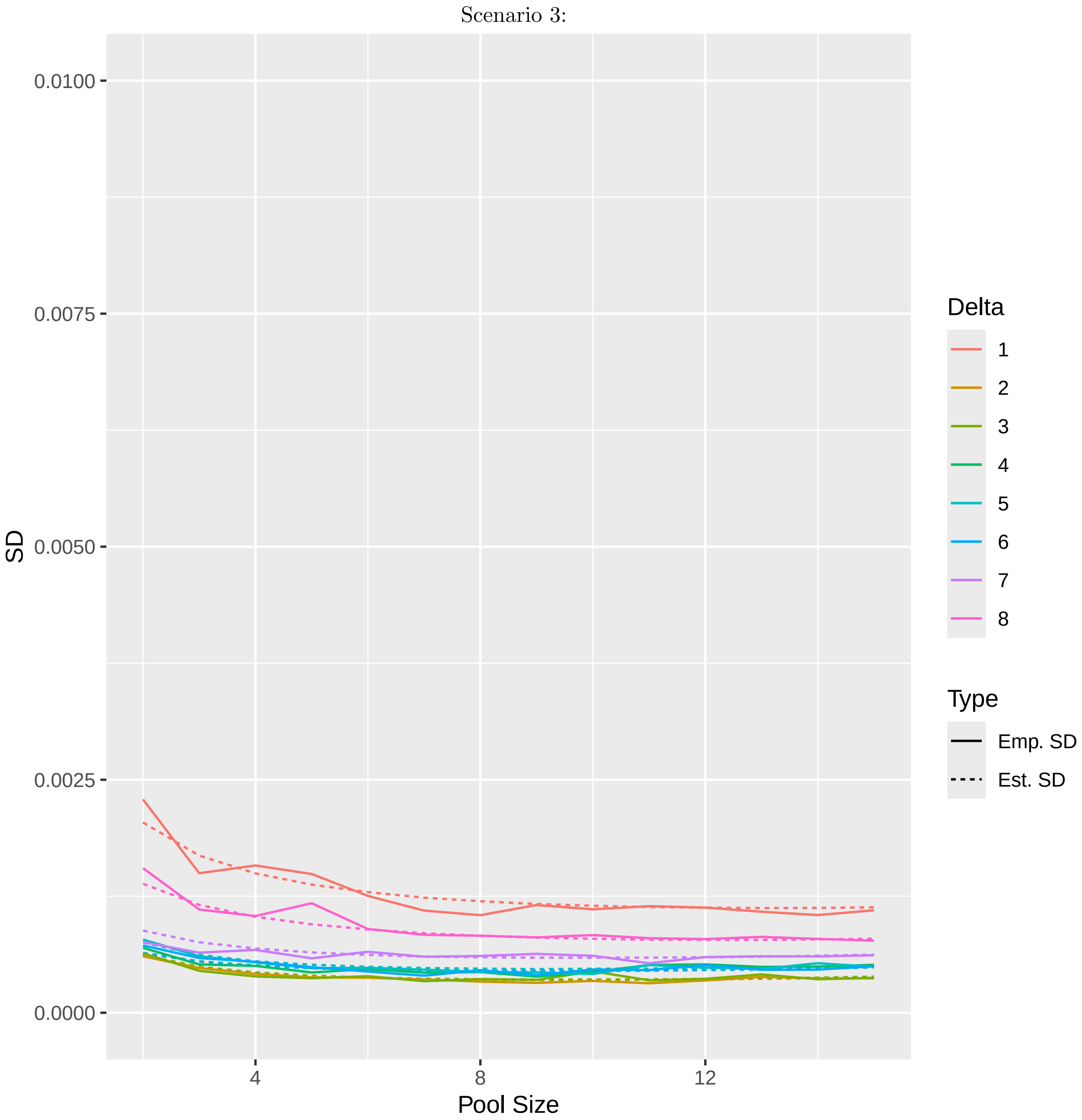

Web Fig. 9: The figure displays the average estimated standard error from Louis's method and the standard deviation of the sample of point estimators for $\hat{\boldsymbol{\delta}}$ from scenario 3 as a function of pool size. From 1 to 8, the position numbers correspond to $\boldsymbol{y} = (0, 0, 0)$, $(1, 0, 0)$, $(0, 1, 0)$, $(1, 0, 1)$, $(1, 1, 0)$, $(0, 1, 1)$ and $(1, 1, 1)$. This is an enlarged version of Figure 2(c).

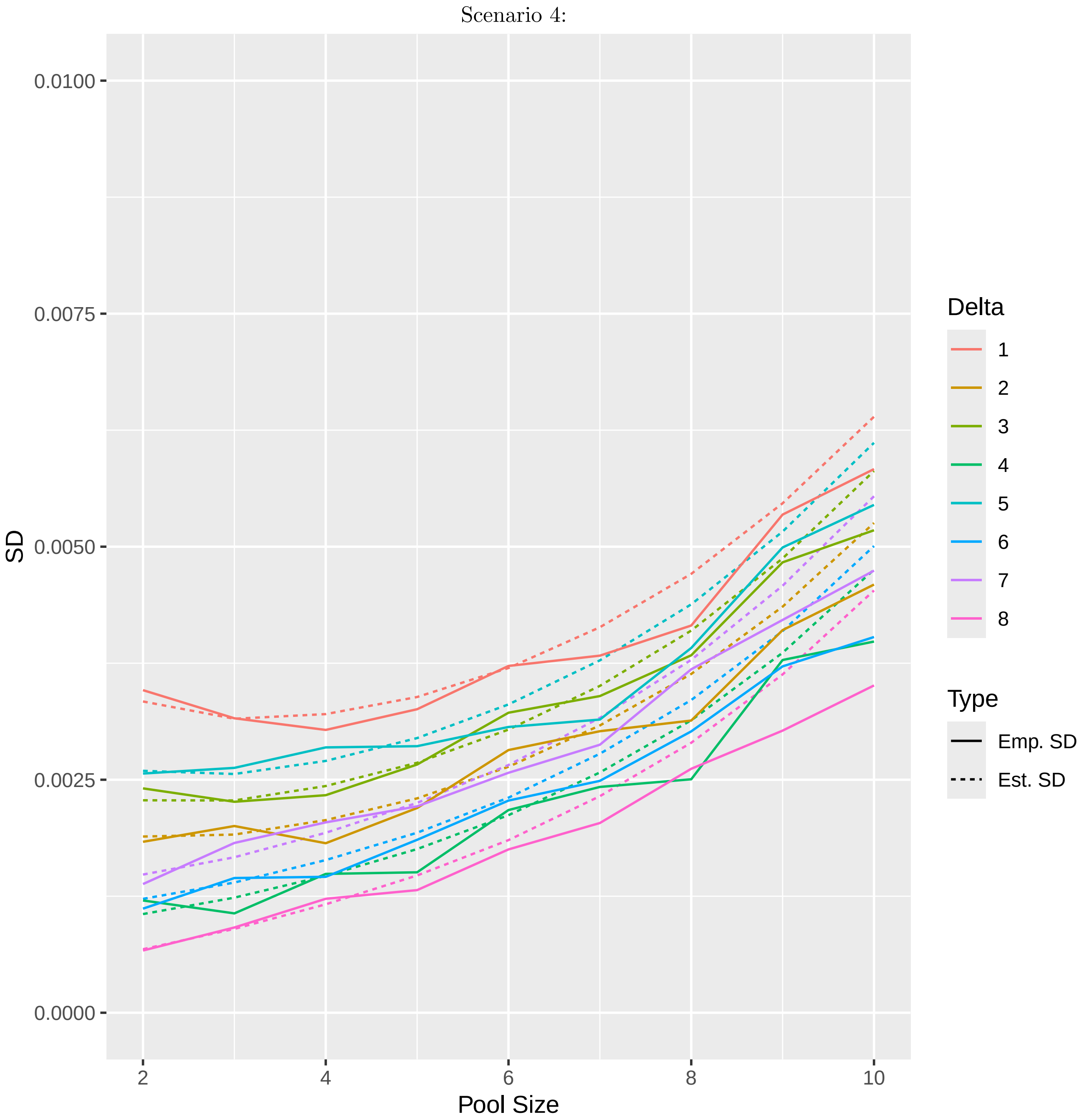

Web Fig. 10: The figure displays the average estimated standard error from Louis's method and the standard deviation of the sample of point estimators for $\hat{\boldsymbol{\delta}}$ from scenario 4 as a function of pool size. From 1 to 8, the position numbers correspond to $\boldsymbol{y} = (0,0,0)$, $(1,0,0)$, $(0,1,0)$, $(1,0,1)$, $(1,1,0)$, $(0,1,1)$ and $(1,1,1)$. This is an enlarged version of Figure 2(d).

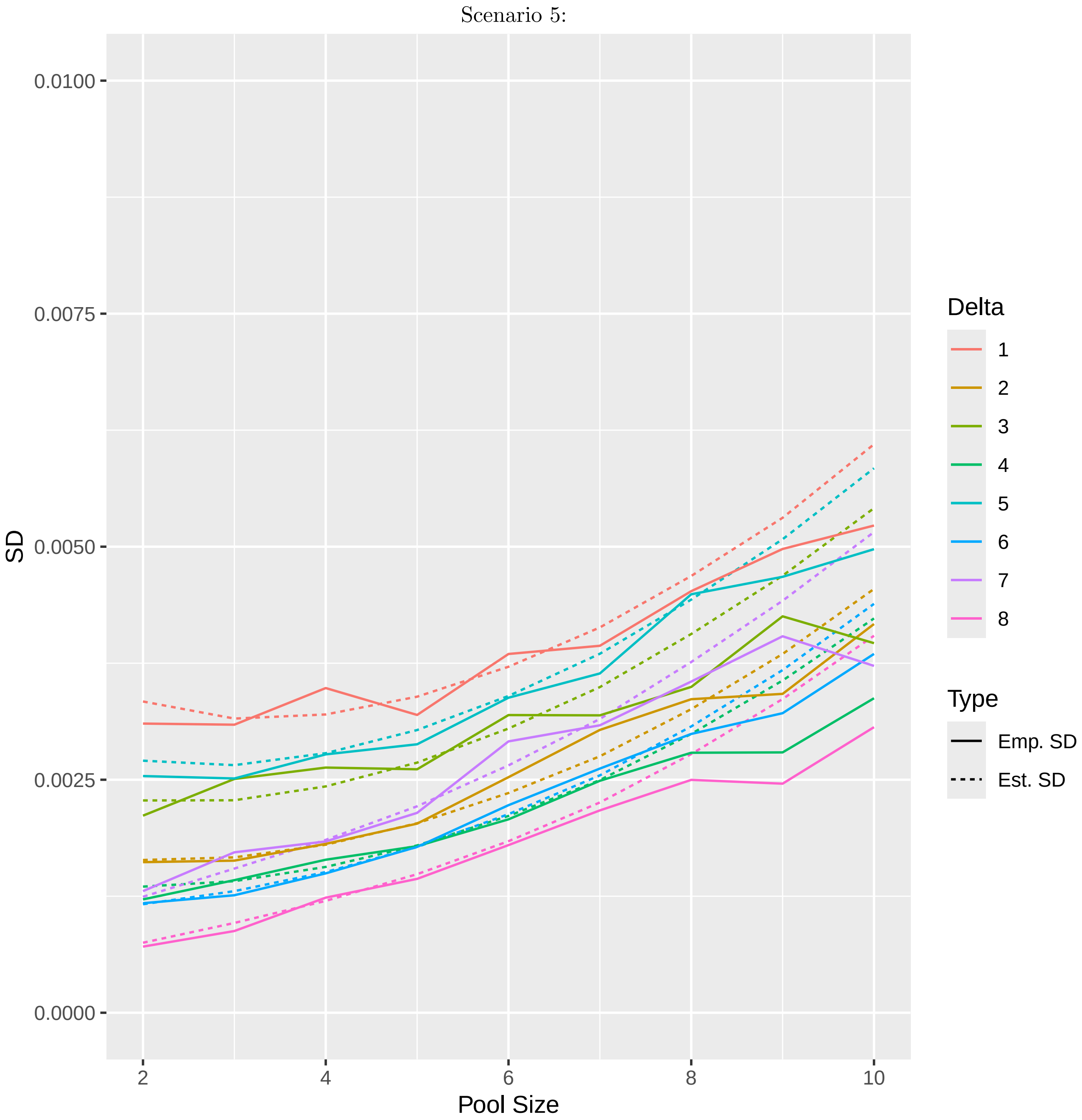

Web Fig. 11: The figure displays the average estimated standard error from Louis's method and the standard deviation of the sample of point estimators for $\hat{\boldsymbol{\delta}}$ from scenario 5 as a function of pool size. From 1 to 8, the position numbers correspond to $\boldsymbol{y} = (0,0,0)$, $(1,0,0)$, $(0,1,0)$, $(1,0,1)$, $(1,1,0)$, $(0,1,1)$ and $(1,1,1)$. This is an enlarged version of Figure 2(e).

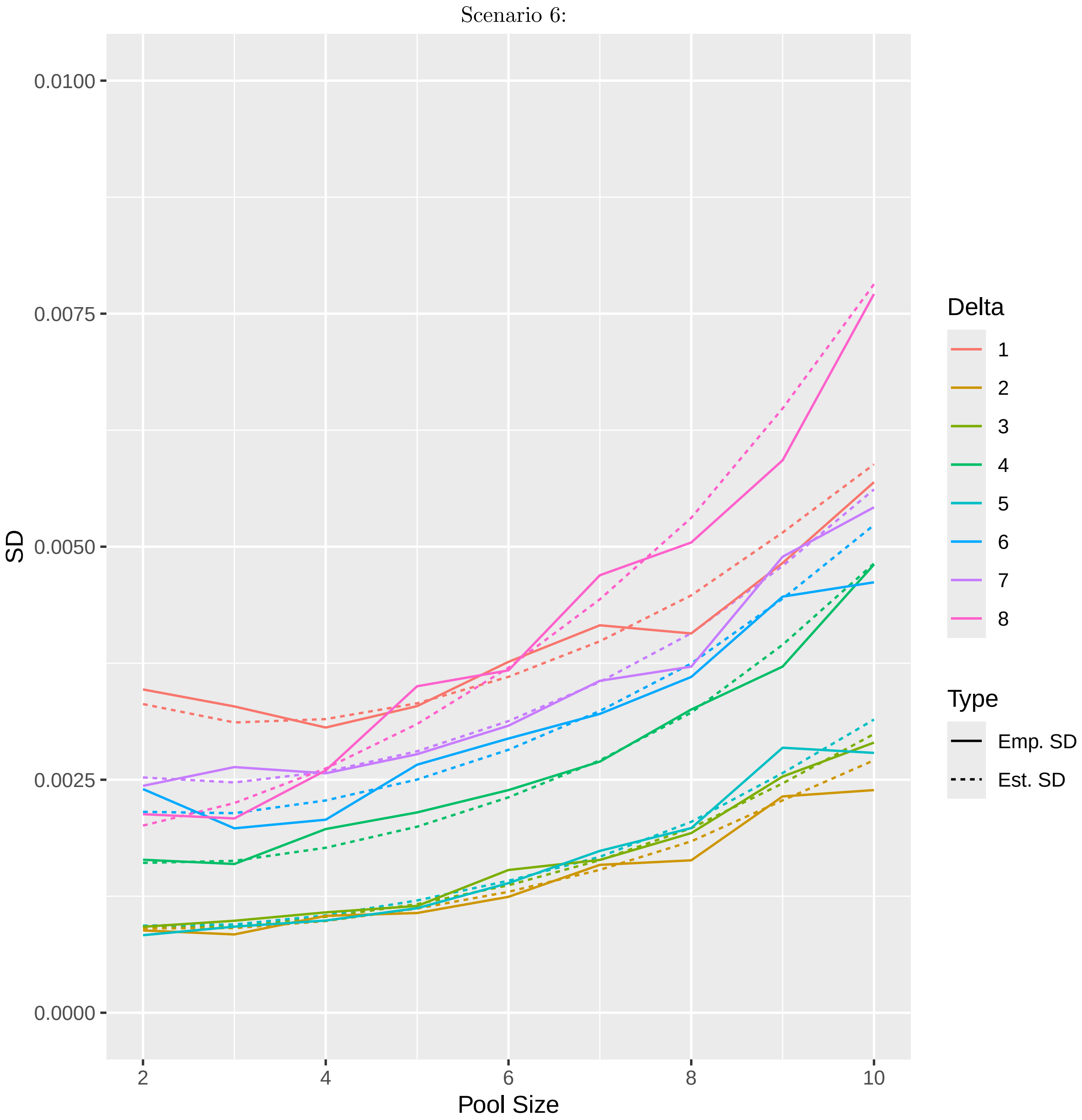

Web Fig. 12: The figure displays the average estimated standard error from Louis's method and the standard deviation of the sample of point estimators for $\hat{\boldsymbol{\delta}}$ from scenario 6 as a function of pool size. From 1 to 8, the position numbers correspond to $\boldsymbol{y} = (0,0,0)$, $(1,0,0)$, $(0,1,0)$, $(1,0,1)$, $(1,1,0)$, $(0,1,1)$ and $(1,1,1)$. This is an enlarged version of Figure 2(f).

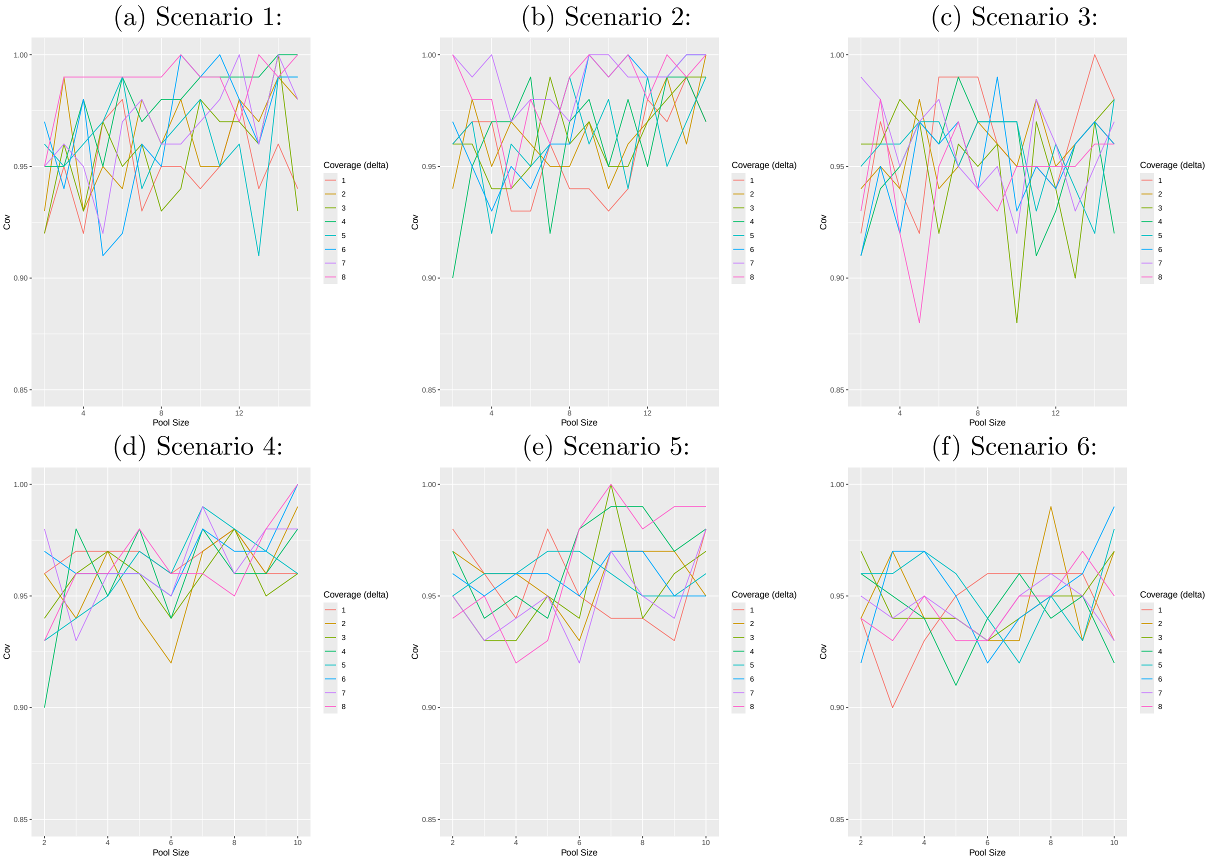

Web Fig. 13: The figure displays the empirical coverage probabilities for 95% confidence intervals for $\hat{\boldsymbol{\delta}}$ as a function of pool size. From 1 to 8, the position numbers correspond to $\boldsymbol{y} = (0,0,0)$, $(1,0,0)$, $(0,1,0)$, $(1,0,1)$, $(1,1,0)$, $(0,1,1)$ and $(1,1,1)$. The figure panels correspond to scenarios 1-6 from top left (1) to bottom right (6).

(a) Scenario 1: (b) Scenario 2: (c) Scenario 3:

(d) Scenario 4: (e) Scenario 5: (f) Scenario 6:

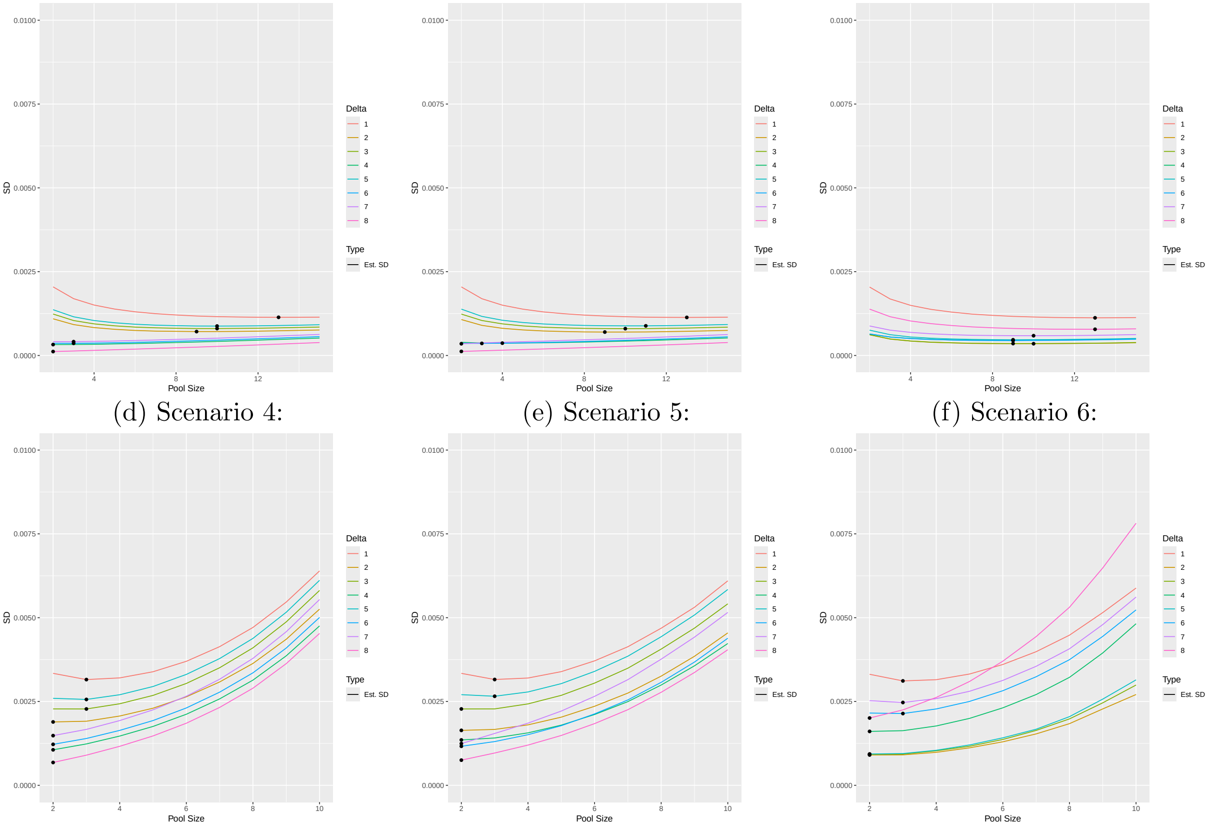

Web Fig. 14: The figure displays the average standard errors from Louis's method for $\hat{\boldsymbol{\delta}}$ as a function of pool size. Black dots indicate the pool size which minimizes the standard error. From 1 to 8, the position numbers correspond to $\boldsymbol{z} = (0,0,0)$, $(1,0,0)$, $(0,1,0)$, $(1,1,0)$, $(0,0,1)$, $(1,0,1)$, $(0,1,1)$ and $(1,1,1)$. The figure panels correspond to scenarios 1-6 from top left (1) to bottom right (6).

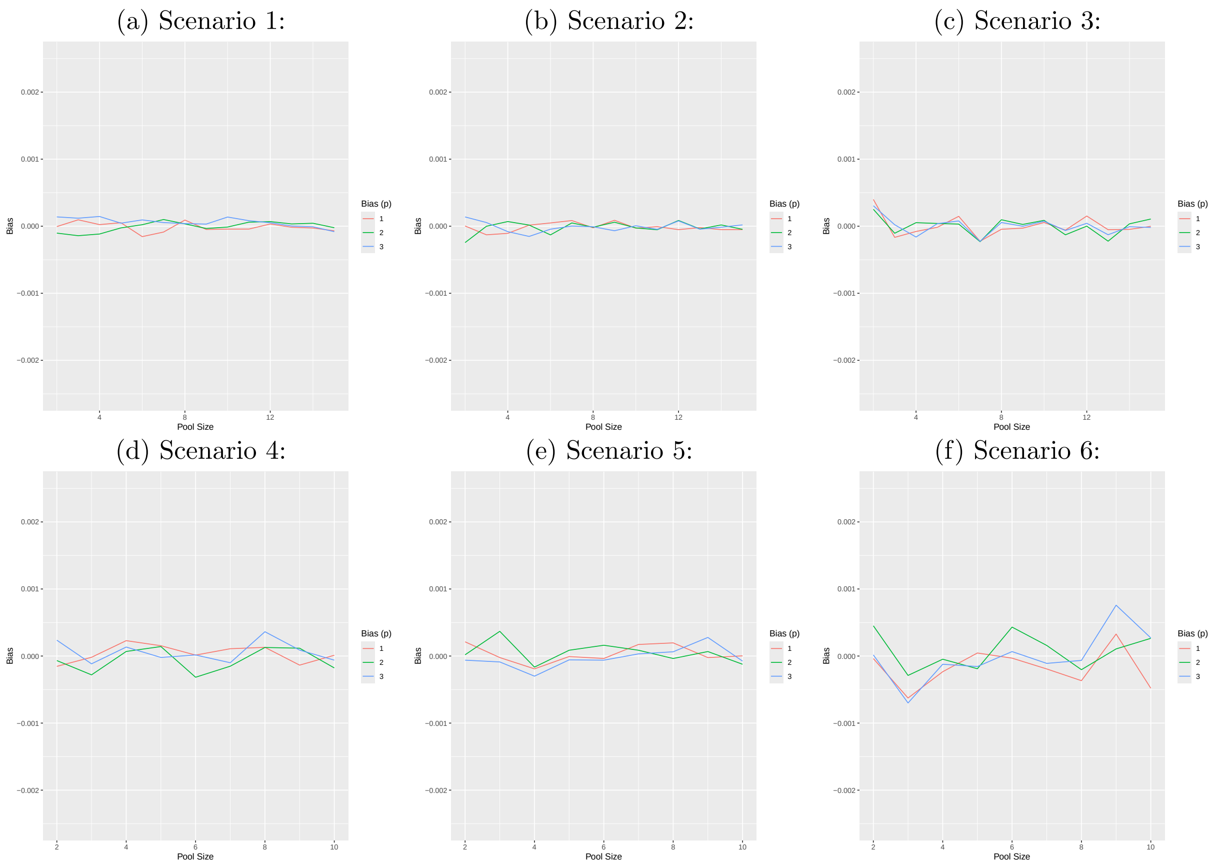

Web Fig. 15: The figure displays the average empirical bias of the estimator $\hat{\boldsymbol{p}}$ as a function of pool size. The figure panels correspond to scenarios 1-6 from top left (1) to bottom right (6).

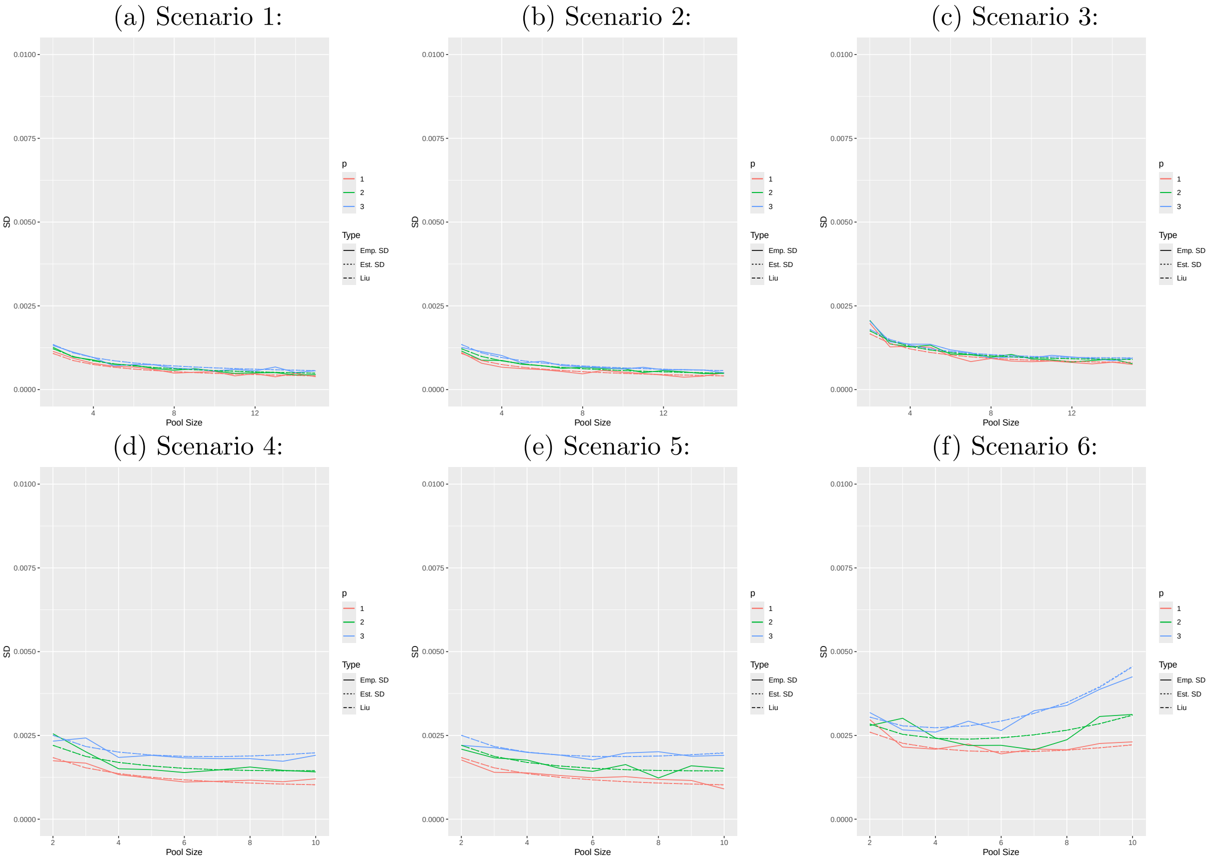

Web Fig. 16: The figure displays the average estimated standard error from Louis's method, the sample standard deviation of the point estimators for $\hat{\boldsymbol{\delta}}$ as a function of pool size and the standard error calculated using Liu et. al's method for singlexplex assays. From 1 to 8, the position numbers correspond to $\boldsymbol{z} = (0,0,0)$, $(1,0,0)$, $(0,1,0)$, $(1,0,1)$, $(1,1,0)$, $(0,1,1)$ and $(1,1,1)$. The figure panels correspond to scenarios 1-6 from top left (1) to bottom right (6).

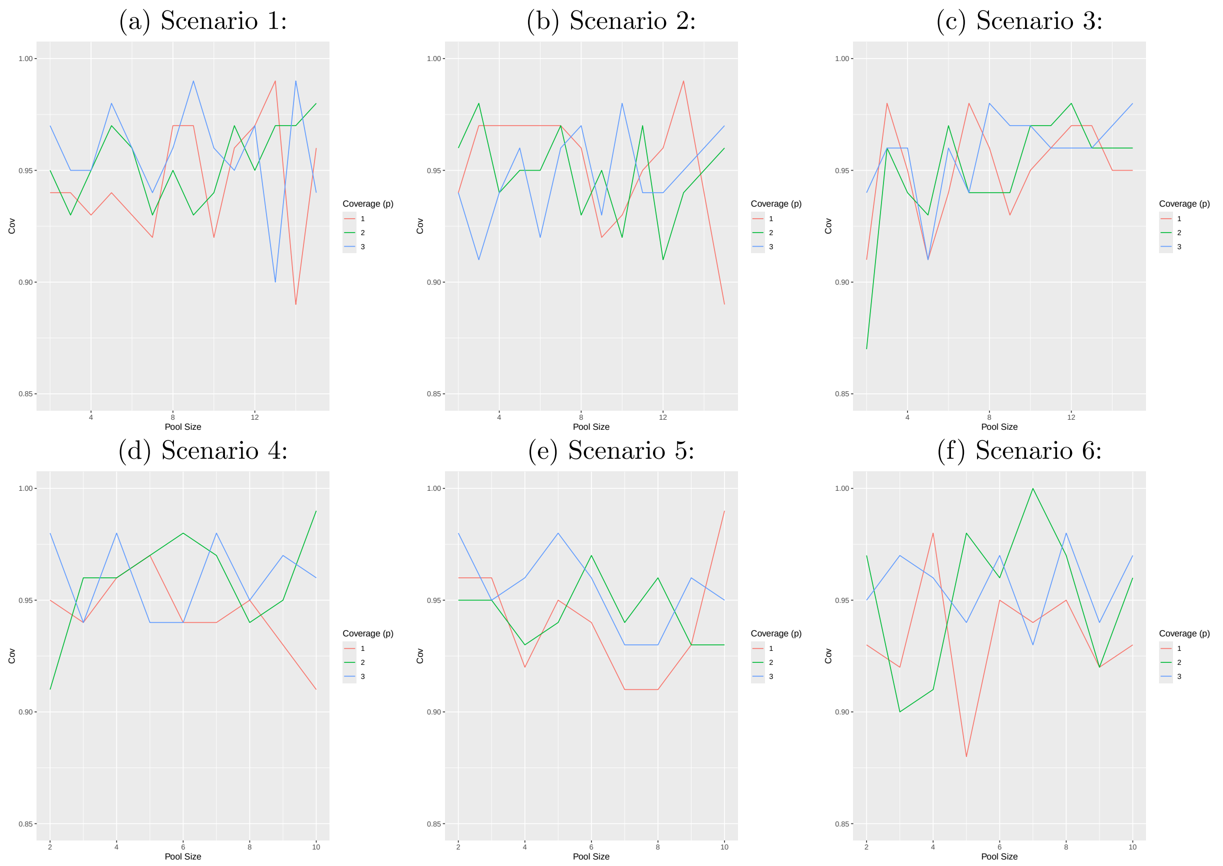

Web Fig. 17: The figure displays the empirical coverage probabilities for 95% confidence intervals for $\hat{\boldsymbol{p}}$ as a function of pool size. The figure panels correspond to scenarios 1-6 from top left (1) to bottom right (6).

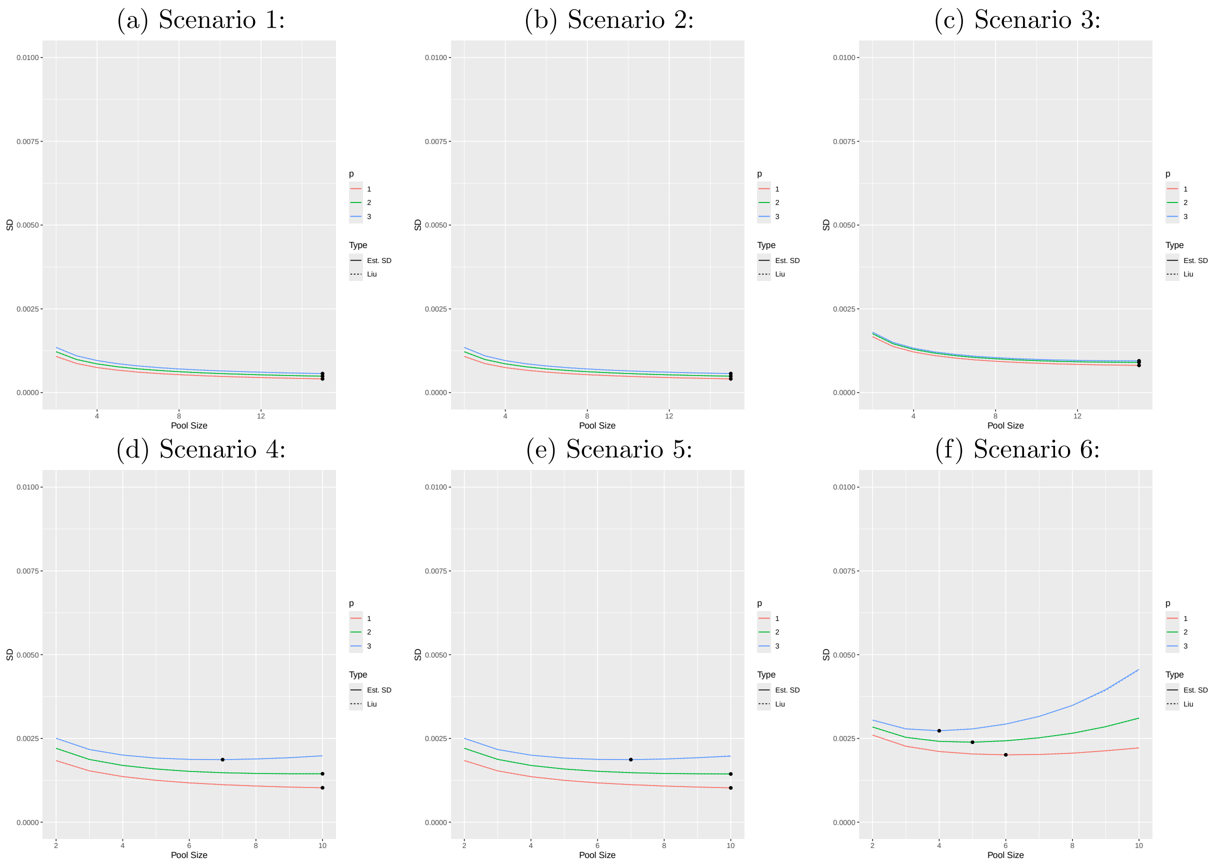

Web Fig. 18: The figure displays average estimated standard error from Louis's method for $\hat{\boldsymbol{p}}$ as a function of pool size. Black dots indicate the pool sizes which minimizes the standard error. The figure panels correspond to scenarios 1-6 from top left (1) to bottom right (6).
